# Understanding the bias of mobile location data across spatial scales and over time: A comprehensive analysis of SafeGraph data in the United States

**DOI:** 10.1371/journal.pone.0294430

**Published:** 2024-01-19

**Authors:** Zhenlong Li, Huan Ning, Fengrui Jing, M. Naser Lessani

**Affiliations:** Geoinformation and Big Data Research Laboratory, Department of Geography, The Pennsylvania State University, University Park, Pennsylvania, United States of America; University of Cyprus, CYPRUS

## Abstract

Mobile location data has emerged as a valuable data source for studying human mobility patterns in various contexts, including virus spreading, urban planning, and hazard evacuation. However, these data are often anonymized overviews derived from a panel of traced mobile devices, and the representativeness of these panels is not well documented. Without a clear understanding of the data representativeness, the interpretations of research based on mobile location data may be questionable. This article presents a comprehensive examination of the potential biases associated with mobile location data using SafeGraph Patterns data in the United States as a case study. The research rigorously scrutinizes and documents the bias from multiple dimensions, including spatial, temporal, urbanization, demographic, and socioeconomic, over a five-year period from 2018 to 2022 across diverse geographic levels, including state, county, census tract, and census block group. Our analysis of the SafeGraph Patterns dataset revealed an average sampling rate of 7.5% with notable temporal dynamics, geographic disparities, and urban-rural differences. The number of sampled devices was strongly correlated with the census population at the county level over the five years for both urban (r > 0.97) and rural counties (r > 0.91), but less so at the census tract and block group levels. We observed minor sampling biases among groups such as gender, age, and moderate-income, with biases typically ranging from -0.05 to +0.05. However, minority groups such as Hispanic populations, low-income households, and individuals with low levels of education generally exhibited higher levels of underrepresentation bias that varied over space, time, urbanization, and across geographic levels. These findings provide important insights for future studies that utilize SafeGraph data or other mobile location datasets, highlighting the need to thoroughly evaluate the spatiotemporal dynamics of the bias across spatial scales when employing such data sources.

## 1. Introduction

Mobile location data has become increasingly important for understanding human mobility patterns in contemporary society. Modern smartphones are equipped with highly sensitive Global Positioning System (GPS) receivers that can provide accurate location data to installed applications, such as Google Maps [1] and social media platforms including Twitter, Facebook, and Instagram [2]. Such location data has become a vital source of geospatial big data for human mobility studies, allowing researchers to gain insights into travel trajectories, activity patterns, and behavior across large geographic areas with a high level of granularity [3, 4]. Several commercial data companies started to provide mobile location data including SafeGraph, Cuebiq, X-mode, and Foursquare, to name a few. These data often do not uniquely identify individuals but rather provide an anonymized overview of their aggregated movement to protect individual privacy while still providing insights into broader patterns of human mobility. For example, mobile location data may be anonymized and aggregated at the neighborhood or census block level, rather than at the level of individual users.

SafeGraph Patterns data [5], or Advan Patterns since 2023 [6], has emerged as one of the most frequently utilized sources of mobile location data in academic research across multiple domains, particularly in the realm of urban sciences, public health, consumer behaviors, and environmental science. For instance, in urban sciences, the data has been used for analyzing human mobility patterns within and between various regions [7], evaluating transportation infrastructures and planning [8], analyzing transportation equity and socioeconomic disparities [9, 10], and assessing the accessibility of bus rapid transit [11]. Similarly, SafeGraph data has been extensively used in public health studies, including tracking the spread of infectious diseases [12, 13], monitoring social distancing behaviors, examining the effectiveness of control measures during the COVID-19 pandemic [14, 15], and investigating the impacts of non-pharmaceutical intervention [16]. In addition, such datasets play a pivotal role in environmental sciences, such as understanding factors influencing long-term park visitation [17], estimating visitors’ demographic status and their patterns in national parks [18, 19], and examining how urban socio-physical system impact on the resilience of cities [20]. Furthermore, numerous studies used SafeGraph data for marketing and consumer behavior research, especially to understand how people move and interact with businesses and commercial areas and predict consumer behaviors [21, 22].

One potential source of harm associated with the use of big data in research and applications is the risk of incorporating implicit biases into analyses that impact the accuracy and reliability of research findings [23]. Due to its nature as big data, mobile location data such as SafeGraph is particularly susceptible to sampling bias because deriving mobility datasets from mobile devices involves multiple successive sampling procedures, from the population to mobile phone owners and app users [24]. Sampling bias refers to the discrepancy between a sample and the population from which it was collected. It is a systematic error that cannot be alleviated by simply increasing the size of the sample [25]. In the context of SafeGraph data, ’sample’ refers to the panel of devices compiled within the dataset, while the ’population’ denotes the entirety of the U.S. population, and the bias can arise from multiple dimensions. Sampling bias can result in certain demographic and socioeconomic groups being overrepresented or underrepresented. For instance, if the data is collected only from users who have downloaded particular apps, it may not be representative of the entire population. In addition, the rate of access to smartphones can vary across diverse age groups and genders [26] and mobile location data is collected from individuals who have opted in to share their location data. Location is another dimension of the sampling bias, as the availability and quality of mobile location data can vary across various geographic regions. In certain geographic areas, the bias can result from a range of factors, including weaker mobile signals or a small number of cell towers or urban-rural settings. In addition, the popularity of certain location-based apps or services may also contribute to disparities in the types and quantity of location data collected in distinct geographical zones [27]. Finally, the bias is likely to change over time as data collection at certain times of the day or week or at different seasons may influence the detection of specific mobility patterns. For example, the frequency and regularity of data collection, in which data collected during peak hours, potentially overestimated the number of individuals using transportation infrastructure, while data collected during off-peak hours possibly underestimated the rate of mobility.

To alleviate concerns over potential data bias, SafeGraph provides a preliminary assessment of bias in its Patterns datasets, revealing high Pearson coefficients (>0.96) between the number of sampled devices and the population at the state and county level [28]. Similarly, aggregated tracked devices in the SafeGraph data exhibit an exceptionally strong association with the population at the national level (>0.99) for different demographic groups, including race, education attainment, and household income. Wang et al. [29] investigated the association between healthcare visits and neighborhood socioeconomics during the COVID-19 pandemic, and found that the sampling rates were balanced overall at the state level in North Carolina. Coston et al. [30] introduced external datasets to audit the bias of SafeGraph data. They examined the voter turnout data of North Carolina’s 2018 general election and found that older and non-white voters appear to be less captured in SafeGraph visits in poll locations. Their study demonstrated a workflow to leverage administrative data for mobility bias detection, yet the accessibility of such data is limited since rare events involving dramatic foot traffic collect demographic information. Although these studies provide useful insights into SafeGraph data bias, they do not provide a comprehensive evaluation of the bias or analyze the spatial and temporal aspects of identified bias at different spatial scales. In light of the potential impact of bias on research outcomes, it is imperative that researchers make a concerted effort to understand and mitigate bias in mobile location data such as SafeGraph and maintain the highest standards of rigor and accuracy in their studies. While there is no universally accepted standard for validating mobile location data [24], conducting a systematic analysis of data bias at multiple geographical levels over a longitudinal study would be beneficial for the research community and improve practical applications of mobile location data.

To address this need, we conducted a comprehensive investigation of bias in mobile location data using the widely used SafeGraph Patterns [6] in the entire United States (US) as our study dataset. The research focused on examining the bias from multiple dimensions including spatial, temporal, urbanization, demographic, and socioeconomic over a five-year period from 2018 to 2022 at multiple spatial scales, including state, county, census tract, and census block group, and at different geographic settings of urban and rural areas. The bias examined covers commonly used demographic and socioeconomic variables such as sex, age, race/ethnicity, income, and educational attainment. The significance of this study lies in four key contributions: 1) a systematic assessment of bias from multiple dimensions across a wide spatiotemporal range (the entire US with monthly data of five years); 2) identification of population bias across a broad range of demographic and socioeconomic variables; 3) spatial and temporal analysis of the quantified bias at multiple spatial scales, and 4) a general analysis framework for evaluating the bias of mobile location data. By systematically documenting the bias at various geographic levels over a five-year period, the findings of this research offer valuable reference for future studies that leverage SafeGraph data or other mobile location datasets.

The remainder of this paper is organized as follows: Section 2 describes the study area and the methodology employed; Section 3 presents and discusses the results; Section 4 delves into the limitations of the presented research; and Section 5 summarizes our findings.

## 2. Data and method

### 2.1 Study area and data

We conducted a nationwide study encompassing the entire US, including Alaska and Hawaii, with geographic units at the census block group, tract, county, and state levels. The boundaries of these geographic units were defined by the US Census Bureau. The Census block group is the smallest publicly available geographic unit for sample data from the decennial census which typically has a population of 600 to 3,000 people. Our analysis utilized the SafeGraph *Panel Overview* data on monthly patterns from 2018 to 2022, spanning five years, sourced from [31]. We extracted the column of *number_devices_residing* for each block group from the panel data as a proxy for residents. This variable indicates the count of distinct devices observed with a primary nighttime location in the specified block group. SafeGraph determines the home location of a device by analyzing data for 6 weeks during nighttime hours (between 6 pm and 7 am) to identify a common nighttime location for the device which is then mapped to a census block group [31].

The socioeconomic and demographic population data were extracted from the American Community Survey (ACS), 5-year estimates [32]. We used the ACS data of 2018 and 2019 at the four geographic levels of block group, tract, county, and state. While ACS employed new boundaries since 2020, SafeGraph data still used block group boundaries from the 2010 Census. As there is no reliable means to align the new boundaries with the previous ones [33–35], we relied on ACS 2019 data to analyze bias for 2020, 2021, and 2022.

We used two distinct sources for the urban-rural classification. To determine the urban classification for block groups, we applied a threshold that assigned an urban label to those areas where more than 50% of the block group’s land area fell within the urban polygons defined by the US Census Bureau. These polygons were delineated at the Census block level, using housing density as the primary criterion [36]. For tract level, we employed the urban-rural classification scheme from USDA ERS (2020a), which classified tracts with centroids located within Census urban polygons as urban [36]. For the county level, we used the classification scheme from USDA ERS (2020b), which contains two major categories (metro and nonmetro) and nine sub-categories based on population, urbanization degree, and adjacency to metro areas. The metro counties were classified as urban and the nonmetro counties were categorized as rural in this study.

### 2.2 Analyze bias for the total population

In this analysis, we focused on the representativeness of the total population of the SafeGraph data without considering different population groups. Specifically, we used sampling rate to analyze the spatial and temporal bias of the data for the whole population across five geographic levels (block group, tract, county, state, and nation) in the US. To compute the sampling rate for a specific geographic level, we summed the number of residing devices for each geographic unit and then divided this sum by the corresponding population of the unit. We hypothesized that data without bias should exhibit a consistent sampling rate across space, and data without temporal bias should demonstrate a constant sampling rate over time.

To examine the temporal trend of the bias and identify the potential urban-rural disparities, we computed the five-year (2018–2022) monthly nation level sampling rate and its associated urban-rural classification. Violin charts were further generated to display the distribution of the sampling rate in each month in urban and rural areas. Furthermore, to examine the geographic disparities of the bias, we further mapped the sampling rates in four geographic levels (block group, tract, county, and state) in the US. These maps aim to provide SafeGraph data users with an intuitive visual understanding of the representativeness of SafeGraph data for the entire population at different geographic levels.

### 2.3 Analyze socioeconomic and demographic bias for different population groups

In this analysis, we focused on examining the data’s representativeness of different population groups at the county and state levels. Specifically, we investigated whether the tracked mobile devices provided by SafeGraph are evenly distributed among 23 different population groups classified with demographic and socioeconomic variables. These variables cover five categories, including age, gender, race/ethnicity, education, and income (Table 1).

**Table 1:** Definition of the selected demographic and socioeconomic variables.

Category	Population Groups	Definition
**Population**	–	Total population in a geographic unit
**Gender**	Female	Population of female
	Male	Population of male
**Age**	15–17^1^	Population in the age range
	18–24	
	25–34	
	35–44	
	45–54	
	55–64	
	> 65	
**Race/ethnicity**	White	Population of White
	Black	Population of Black or African American
	Hispanic	Population of Hispanic or Latino
	Asian	Population of Asian
Education Attainment^2^	NoSchool	Population ≥ 25 less than high school
	HighSchool	Population ≥ 25 high school graduates or equivalency
	NoCollege	Population ≥25 less than high school or high school graduate or equivalency
	Bachelor	Population ≥ 25 have a Bachelor’s degree
	Graduate	Population ≥ 25 have a master, doctorate, or professional degree
Income^3^	< 20K	Households in the income range
	20K – 30K	
	30K – 50K	
	50K – 100K	
	>100K	

^1^SafeGraph does not track children (age<16)’s devices, but the ACS data contains age group of 15–17 only. While the devices of 15 years old were not tracked, we keep this group in this study as it still has a large overlap with the SafeGraph data.

^2^The proportion is calculated by dividing the population ≥25.

^3^The proportion is calculated by dividing the total household.

We hypothesized that data without bias should have the same sampling rate among different population groups. Subsequently, any differences in the sampling rate are viewed as bias. Following SafeGraph [28] and Wang et al. [29], we adopted an aggregation-based bias inspection approach to assess the bias among population groups (denoted as *g*) at county and state levels. Specifically, we assumed that in a block group (denoted as *c*), the sampled devices (*D*_*c*_) have the same demographic characteristics as that block group, meaning that they share the same proportions of each population group (*p*_*g_c*_). Next, we aggregated the sampled devices of each population group to the county (or state) level to calculate the county (or state) level bias ($\sum_{c=1}^{n}D_{c}\cdot p_{g\_c},n$ is the number of block groups of the county or state). Specifically, for the population group *g*, the proportion of sampled devices relative to the total number of devices in the county or state ($P_{g}^{safegraph}$) can be obtained by Eq (1), where $\sum_{c=1}^{n}D_{c}$ is the total number of sampled devices. Similarly, the proportion of *g* to the total county or state population ($P_{g}^{census}$) can be obtained by Eq (2), where *P*_*c*_ is the population of block group *c* and $\sum_{c=1}^{n}P_{c}$ is the total county (or state) population. Ideally if the data has no bias, $P_{g}^{safegraph}$ should equal to $P_{g}^{census}$, indicating their ratio is 1. By computing the difference between the actual ratio and 1 for each population group, we can estimate whether the sampled device is evenly distributed among block groups or not as illustrated in Eq (3). A bias value far from 0 indicates high sampling bias, with positive values indicating over-representing (over-sampling) a specific population group and negative values indicating underrepresenting (under-sampling) a specific population group. For example, a bias value of 0.05 for a specific population group indicates that this population group is 5% overrepresented in the data, while a bias value of -0.05 indicates 5% underrepresentation.


$$p_{g}^{safegraph}=\frac{\sum_{c=1}^{n}D_{c}\cdot p_{g\_c}}{\sum_{c=1}^{n}D_{c}}$$ (1)

$$p_{g}^{census}=\frac{\sum_{c=1}^{n}P_{c}\cdot p_{g\_c}}{\sum_{c=1}^{n}P_{c}}$$ (2)

$$bias=\frac{p_{g}^{safegraph}}{p_{g}^{census}}-1$$ (3)

To examine spatial and temporal patterns of the quantified biases, we mapped the bias of each population group at the county and state levels for each of the five years. The maps were designed to provide an intuitive visual representation of the socioeconomic and demographic bias across different spatial scales and over time for the US. We further created a heatmap to visualize the monthly trend of the bias of each population group from 2018 to 2022. The goal was to provide a clearer understanding of the disparities and changes in bias over time, and to identify areas and groups that may require additional attention in terms of data analysis.

Note that SafeGraph excluded children of age <16, so the sampling rate derived from its datasets is roughly an “adult sampling rate.” However, we stick to the term “sampling rate” since it conveys an intuitive and clear idea that it is a ratio of the sampled devices to the total population. Furthermore, in reality, children usually stay with guardians or caregivers, not completely separating from adults. So, human mobility studies usually cannot exclude children. SafeGraph datasets excluded children’s devices, but not children themselves. According to Sun et al. [37], about 75% of children have a smartphone between 11 and 15 years old; such age group takes up about 6.5% of the total US population. Therefore, about 4.9% (6.5%×75%) of the population’s devices were excluded. In addition, children <15 compose about 18% of the US population [38]. It is worth noting this issue for a better interpretation of our analysis.

## 3. Results and discussion

### 3.1 Sampling rate for the total population

#### 3.1.1 Temporal dynamics

**Fig 1:**
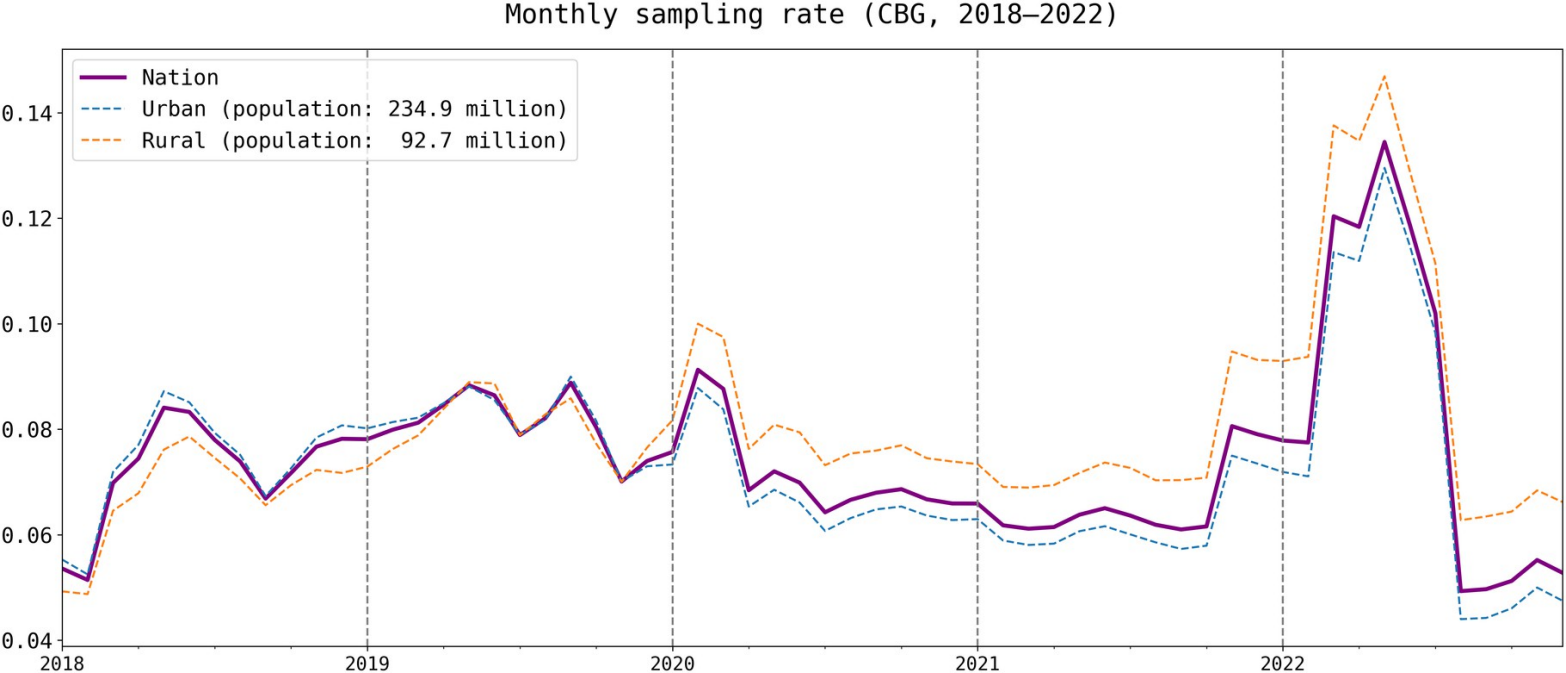
Monthly sampling rate across three categories of urban, rural, and nation from 2018 to 2022. CBG: Census block group.

The overall temporal trend of the monthly sampling rate across three categories of urban, rural, and the nation from 2018 to 2022 is shown in Fig 1. The national sampling rate is obtained by dividing the total number of devices of all block groups by the US population. SafeGraph used multiple criteria to identify the home block group of a device; one criteria is determining which block group a device spent the most nights over the past six weeks. As a result, the devices tracked by the SafeGraph panel are dynamic, varying from block group to national level, due to the addition of new devices and the relocation of residents. For the urban and rural categories, the sampling rates are computed by dividing the total number of devices of block groups classified as urban or rural by the respective population counts. Throughout the five-year period, the sampling rate for all three categories displayed notable fluctuations, ranging from 4.5% to 14.5% with an average of 7.5%. The average sampling rates for the entire US across the five years were 7.2%, 8.1%, 7.2%, 6.6%, and 8.5%, respectively, from 2018 to 2022.

The rates show a significant increase between February and June of 2018, followed by a decline until September, and then another increase until May 2019. The trend also reveals a dramatic decline in March 2019 following the COVID-19 outbreak in the US. After the outbreak, the government released several travel restriction measures, resulting in a significant decline in population movement [7, 9]. The reduced human movement might have resulted in lower sampling rates as SafeGraph data is collected from mobile device applications, which rely on users opting to share their location data while using the applications. The sampling rates continued to decline for an extended period until October 2021, when a significant recovery occurred. The recovery trend reached its highest peak (13.4%) in May 2022, followed by a sharp decline to the lowest point (4.9%) in July 2022. This dramatic change may relate to data provider disruption in May 2022 [34].

The sampling rates for the nation, urban, and rural areas exhibit consistent trends, with similar rates observed for the nation and urban areas. It is worth noting that prior to late 2019, the rural population was generally underrepresented as indicated by the lower sampling rate. However, this trend reversed after late 2019, with the rural population becoming overrepresented and urban population become underrepresented. The disparities between urban and rural areas also widened. The dynamics of the urban-rural disparities of the sampling rate suggest the importance of understanding the bias in the temporal dimension.

**Fig 2:**
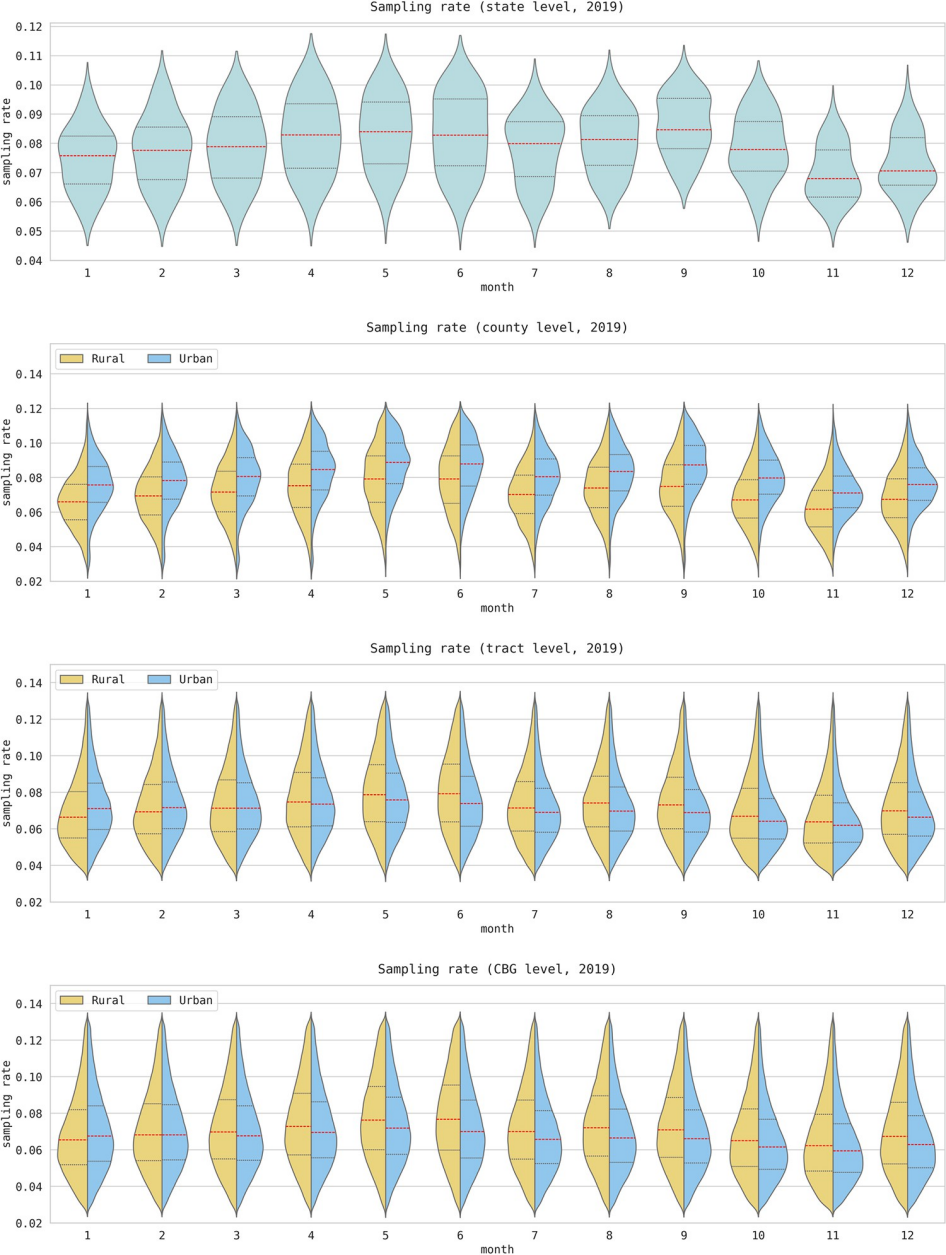
Distribution of the monthly sampling rate at the four geographic levels in 2019. Urban-rural disparities are illustrated for the county, tract, and block group (CBG) levels. The charts for 2018, 2020, 2021, and 2022 are presented in Figs A1, A3-A5 in S1 Appendix.

As the overall temporal trend shows clear temporal bias and urban/rural differences over time, we further calculated the monthly sampling rate for four geographic levels (state, county, tract, and block group) from 2018 to 2022. To compute the sampling rate for a particular geographic level, we first aggregated the number of devices located within each unit and then divided this total by the corresponding population of that unit. At the county, tract, and block group levels, we calculated separate rates for urban and rural areas, and then visualized the results for 2019 using violin charts in Fig 2. The results for 2018, 2020, 2021, and 2022 are presented in Figs A1, A3-A5 in S1 Appendix.

At the county level, the result revealed that the largest rural-urban difference in sampling rate was observed in 2018 and 2019, with a higher sampling rate in urban areas than in rural areas. However, this difference began to decrease in March 2020, and by 2021, there was no discernible difference between urban and rural sampling rates. In 2022, we observed a slight reversal of this pattern, with a slightly higher sampling rate observed in rural areas. At the tract level, we initially observed similar patterns to the county level in 2018. However, the sampling rate became equalized in March, but starting in April 2019, a contradictory pattern occurred: rural areas had a higher sampling rate than urban areas. This pattern continued and gradually intensified throughout the years, with the most dramatic difference observed in 2022. The observed discrepancies in urban-rural sampling rate disparities are likely caused by spatial aggregation and the urban-rural classifications at different geographic levels. The block group level showed similar patterns over the five-year period as the tract level, which may be due to the fact that each tract contains an average of only three block groups.

These findings highlight the importance of analyzing urban-rural disparities in bias for specific geographic levels when using SafeGraph data. For instance, this analysis revealed that in 2019, the data underrepresented the rural population at the county level but overrepresented the rural population at the tract and block group levels. Note that the different classification methods for urban-rural at different geographic levels may affect the bias disparities.

#### 3.1.2 Spatial distribution

**Fig 3:**
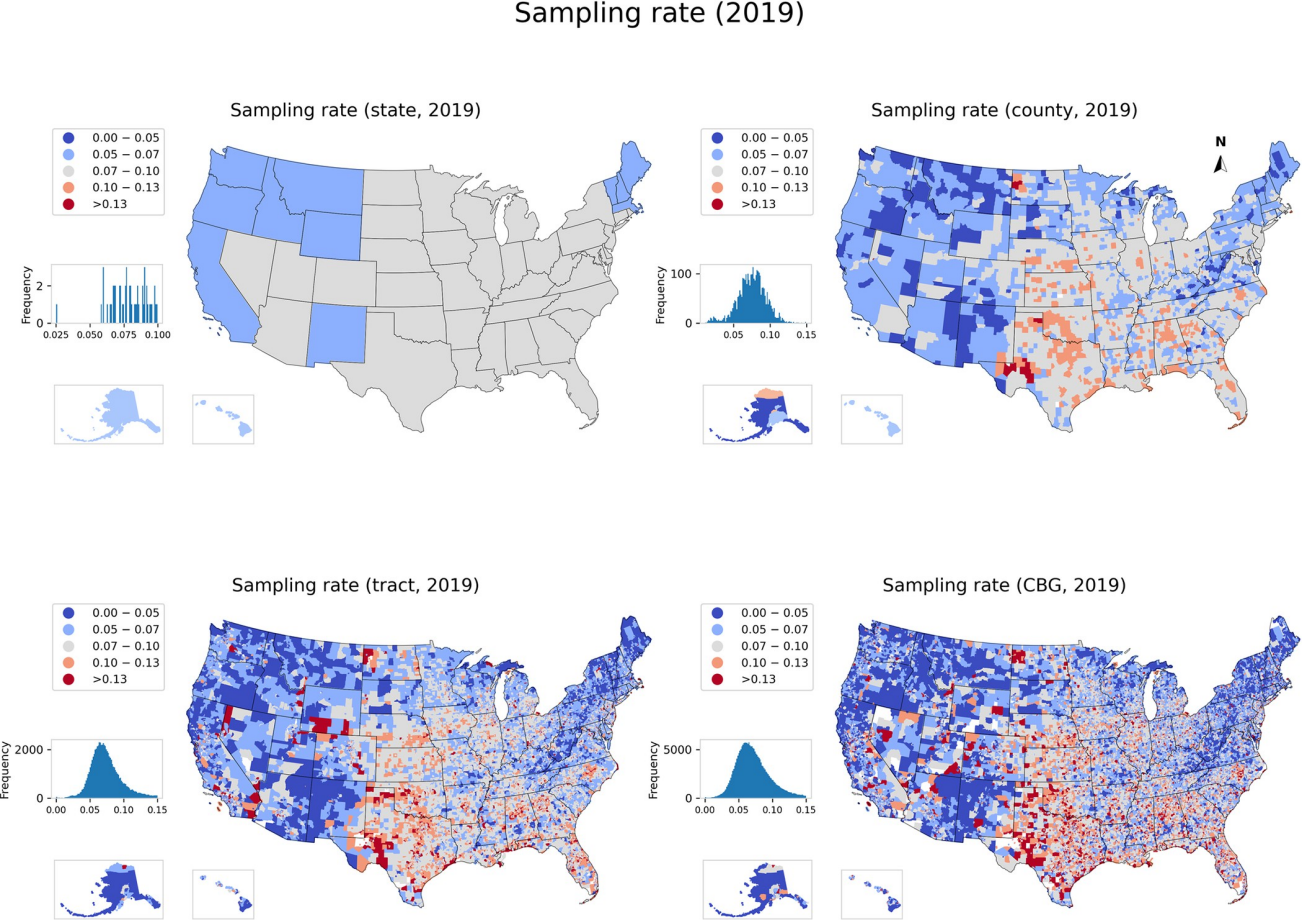
Spatial distribution of the sampling rate in 2019 across four geographic levels, with the frequency of the sampling rate at each level represented as a histogram. The maps for 2018, 2020, 2021, and 2022 are presented in Figs A6, A8-A10 in S1 Appendix.

To examine the geographic disparities in how SafeGraph data represent the whole population, we calculated the yearly sampling rate for each geographic unit at the four geographic levels and mapped the result for 2019 in Fig 3. The maps for 2018, 2020, 2021, and 2022 are presented in Figs A6, A8-A10 in S1 Appendix.

Overall, the nine states in Deep South (i.e., Alabama, Florida, Georgia, Louisiana, Mississippi, North Carolina, South Carolina, Tennessee, and Texas) and three states in the Midwest (i.e., Oklahoma, Kansas, Nebraska) have a higher concentration of areas with higher sampling rates, while the West and Northeast have more areas with lower sampling rates. This pattern was in general consistent over the five years and was clearly illustrated in the maps of county, tract, and block group levels. The maps exhibited a consistent pattern with Fig 1, where the overall sampling rate displayed an upward trend from 2018 (mean: 6.9%) to 2019 (7.1%), decreased in 2020 (7.9%) and 2021 (6.6%) before increasing again in 2022 (8.5%). The maps at the block group level revealed that across five years, the relatively low sampling rates (< 5%, dark blue) were generally concentrated in densely populated areas of the Northeast and West, while the relatively high sampling rates (>10%, dark red) were discretely distributed with spatial clustering in the South. This distribution and trend at the block group level were consistent with those observed at the tract and county levels.

**Fig 4:**
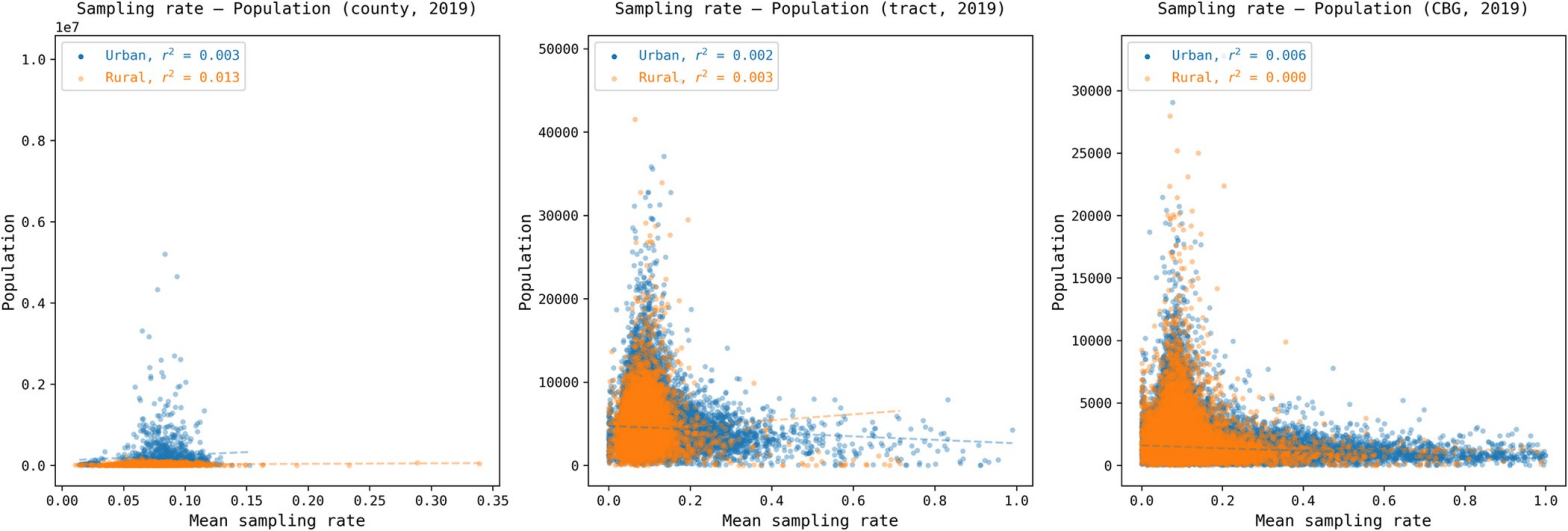
The correlation between the sampling rate and the census population at county, tract, and block group levels with urban/rural classification for 2019. The results for 2018, 2020, 2021, and 2022 are presented in Figs A11, A13-A15 in S1 Appendix.

The maps visually indicated that lower sampling rates tend to be concentrated in densely populated areas of the Northeast and West. To further investigate this observation, we conducted additional analyses to explore whether these geographic disparities are correlated with unit population across various levels of geography. Specifically, for each year and each geographic level (county, tract, and block group), we generated a scatter plot to visualize the relationship between the sampling rate and population. In addition, urban and rural areas were plotted separately to reveal potential urban/rural disparities in such an association. Fig 4 presents the results for 2019, while the results for 2018, 2020, 2021, and 2022 are presented in Figs A11, A13-A15 in S1 Appendix.

Fig 4 illustrates that there is insignificant or no association between the sampling rate and the population at all geographic levels, with *r*^2^ ranging from 0.001 to 0.02 for both urban and rural areas. This pattern is consistent across all five years (Figs A11-A15 in S1 Appendix). The lack of a discernible association between sampling rates and population indicates no systematic bias of sampling rate in these geographic levels or urban/rural settings in terms of population size. Therefore, the geographic disparities depicted in the maps (Fig 3) are likely driven by other factors, such as demographic and socioeconomic characteristics of the population, which is examined in Section 3.2.

#### 3.1.3 Association between device count and population

**Fig 5:**
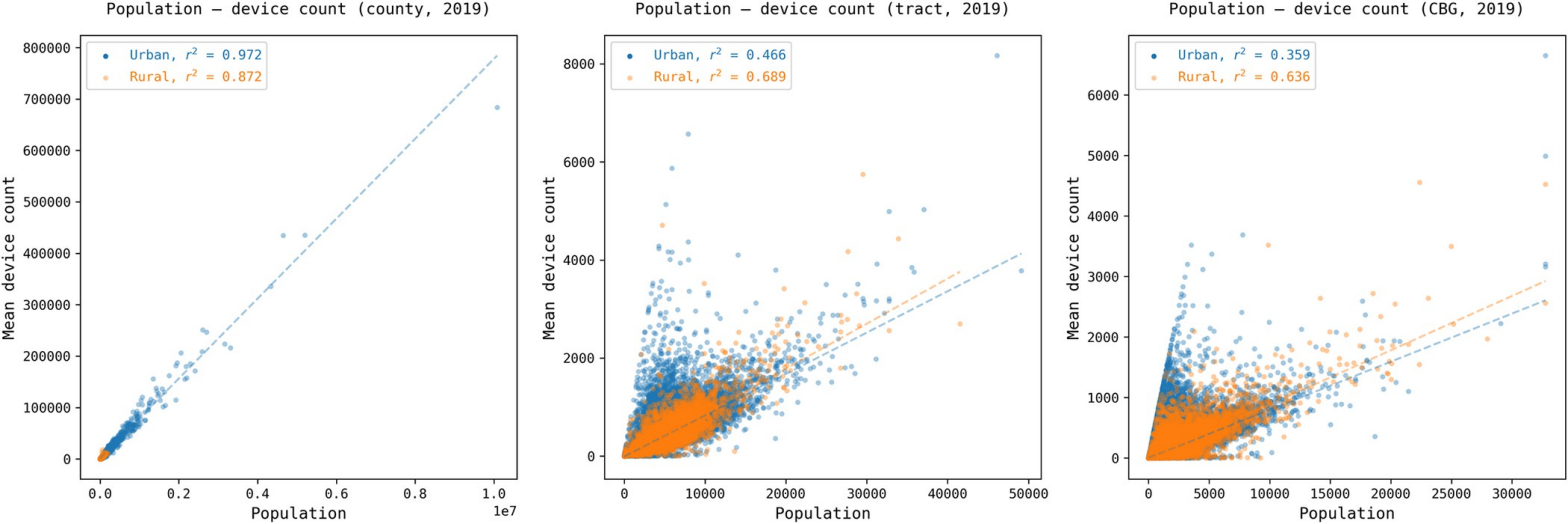
The correlation between the device count and the census population at county, tract, and block group levels with urban/rural classification for 2019. The results for 2018, 2020, 2021, and 2022 are presented in Figs A16, A18-A20 in S1 Appendix.

The above analysis reveals the bias of the sampling rate across different geographic levels and over time. It should be noted that a high sampling rate does not necessarily indicate better representativeness of the population. To assess how well SafeGraph data represents the whole population, we further conducted analyses by creating scatter plots for five years that showed the correlation between the census population and the sampled device count, with urban/rural classification at three geographic levels: county, tract, and block group. The result for 2019 is shown in Fig 5, and the results for 2018, 2020, 2021, and 2022 are presented in Figs A16, A18-A20 in S1 Appendix.

Fig 5 shows the number of sampled devices is strongly correlated with the census population at the county level for both urban and rural counties (*r*^2^ = 0.972, *Pearson r* = 0.986 for urban; *r*^2^ = 0.872, *r* = 0.934 for rural). The association at the tract and block group levels becomes weaker than the county level, but it remains at a moderate level (*r*^2^ > 0.466, *r* > 0.683 for urban tracts; *r*^2^ > 0.689; *r* > 0.830 for rural tracts). This pattern aligns with the uneven geographic distribution of sampling rate at the tract and block group levels, as shown in the previous maps (Fig 3). SafeGraph [28] reported a strong correlation (*r* = 0.966) between the number of sampled devices and the population at the county level using one month data (October 2019), which is consistent with our findings with the 5-year data. However, at the block group level, they reported a much lower correlation coefficient of 0.176 using that one month of data. This finding further highlights the importance of understanding the temporal dynamics of the bias in using SafeGraph data.

Figs A16, A18-A20 in S1 Appendix further revealed that 2019 is generally consistent with the other four years; however, a gradual decline was observed in the representativeness of urban areas, specifically at the tract and block group levels. For example, the *r*^2^ values for urban tracts from 2018 to 2022 were 0.651, 0.466, 0.539, 0.415, 0.375, while the *r*^2^ values for rural tracts remained relatively stable, ranging from 0.678 to 0.735. This finding further underscores the widening urban/rural disparities observed in Figs 1 and 3. Interestingly, at the county level, the data shows slightly better representativeness in urban areas, while at the tract and block group levels, the data perform better in rural areas.

### 3.2 Demographic and socioeconomic bias

#### 3.2.1 Overall demographic and socioeconomic bias among population groups

**Fig 6:**
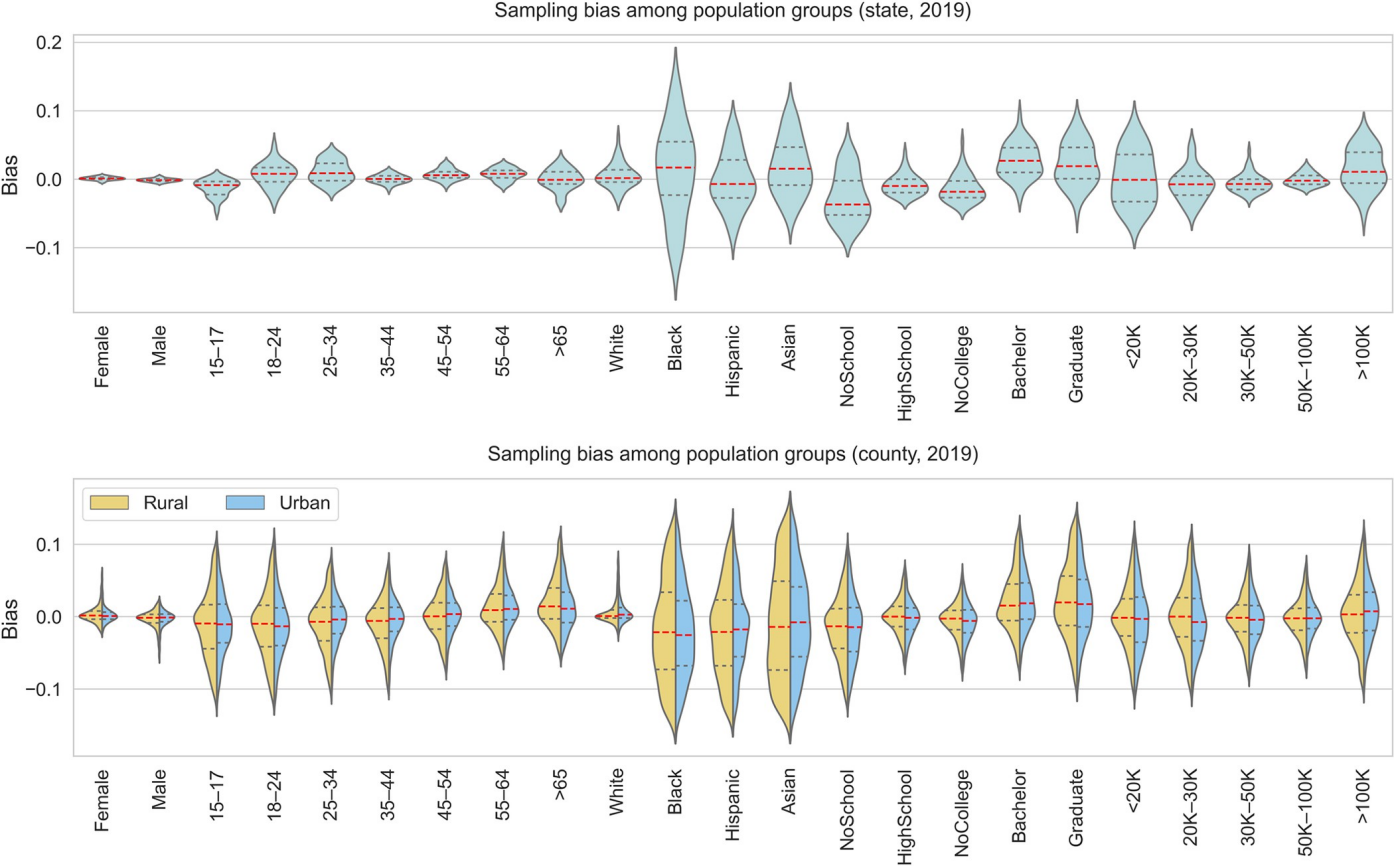
Socioeconomic and demographic bias at state and county levels in 2019. The results for 2018, 2020, 2021, and 2022 are presented in Figs A21, A23-A25 in S1 Appendix.

This analysis aimed to examine the representativeness of SafeGraph data for different population groups across different spatial scales. We calculated the demographic and socioeconomic biases among 23 different population groups with five categories, including age, gender, race/ethnicity, education, and income (Table 1), following the method detailed in section 2.3. Particularly, for each state and county in the US, we calculated the bias for each of the 23 population groups for each year from 2018 to 2022. The frequency distribution of the socioeconomic and demographic bias at the state and county levels in 2019 is presented in Fig 6, while the results for 2018, 2020, 2021, and 2022 are presented in Figs A21-A25 in S1 Appendix. The urban/rural disparities of the bias at the county level are also illustrated in these figures. Additionally, the median, minimum, and maximum bias of population groups for both county and state levels are reported in Tables 2 and 3.

**Table 2:** Sampling biases of population groups at the state level from 2018 to 2022: Median [minimum, maximum] of all states in the US. Since 2019, most biases are in the range of [-0.071, 0.034].

Variable	2018	2019	2020	2021	2022
**Female**	0.001 [-0.009, 0.009]	0.001 [-0.008, 0.011]	0.001 [-0.014, 0.008]	0.002 [-0.008, 0.016]	0.001 [-0.013, 0.011]
**Male**	-0.001 [-0.010, 0.009]	-0.001 [-0.011, 0.009]	-0.001 [-0.009, 0.016]	-0.002 [-0.017, 0.008]	-0.001 [-0.012, 0.013]
**15–17**	**-0.016** [-0.063, 0.016]	-0.009 [-0.051, 0.008]	-0.000 [-0.048, 0.143]	-0.004 [-0.067, 0.068]	-0.010 [-0.052, 0.021]
**18–24**	0.014 [-0.017, 0.075]	0.008 [-0.047, 0.070]	-0.035 [-0.116, 0.059]	-0.015 [-0.097, 0.136]	-0.007 [-0.070, 0.315]
**25–34**	0.024 [-0.015, 0.076]	0.009 [-0.021, 0.061]	-0.014 [-0.043, 0.032]	-0.019 [-0.055, 0.043]	-0.015 [-0.047, 0.046]
**35–44**	0.002 [-0.017, 0.025]	0.000 [-0.037, 0.027]	-0.001 [-0.032, 0.040]	-0.008 [-0.052, 0.048]	-0.010 [-0.066, 0.043]
**45–54**	0.002 [-0.031, 0.016]	0.006 [-0.032, 0.038]	0.016 [-0.043, 0.041]	0.013 [-0.015, 0.042]	0.008 [-0.063, 0.033]
**55–64**	-0.000 [-0.027, 0.025]	0.008 [-0.012, 0.032]	0.022 [-0.003, 0.044]	0.021 [-0.013, 0.057]	0.021 [-0.016, 0.051]
**>65**	-0.010 [-0.077, 0.053]	-0.001 [-0.072, 0.047]	0.019 [-0.055, 0.056]	**0.025** [-0.048, 0.092]	0.024 [-0.061, 0.063]
**White**	-0.008 [-0.099, 0.059]	0.002 [-0.076, 0.076]	0.020 [-0.083, 0.106]	0.020 [-0.111, 0.096]	0.017 [-0.026, 0.085]
**Black**	**0.088** [-0.083, 0.257]	**0.034** [-0.123, 0.549]	-0.046 [-0.188, 0.453]	-0.058 [-0.187, 0.535]	**-0.071** [-0.163, 0.462]
**Hispanic**	0.025 [-0.071, 0.205]	-0.007 [-0.113, 0.109]	**-0.058** [-0.155, 0.068]	**-0.059** [-0.184, 0.165]	-0.022 [-0.163, 0.160]
**Asian**	0.043 [-0.054, 0.218]	0.016 [-0.059, 0.163]	-0.011 [-0.148, 0.098]	-0.035 [-0.162, 0.176]	**-0.071** [-0.177, 0.158]
**NoSchool**	-0.012 [-0.109, 0.144]	**-0.037** [-0.088, 0.100]	-0.045 [-0.114, 0.079]	-0.029 [-0.109, 0.074]	-0.000 [-0.144, 0.101]
**HighSchool**	-0.007 [-0.057, 0.045]	-0.010 [-0.032, 0.073]	0.005 [-0.050, 0.118]	0.011 [-0.036, 0.128]	**0.030** [-0.097, 0.077]
**NoCollege**	-0.007 [-0.081, 0.071]	-0.018 [-0.046, 0.060]	-0.007 [-0.068, 0.082]	0.001 [-0.044, 0.105]	0.024 [-0.119, 0.083]
**Bachelor**	0.014 [-0.069, 0.153]	0.027 [-0.055, 0.094]	0.027 [-0.083, 0.143]	0.012 [-0.095, 0.127]	-0.021 [-0.075, 0.188]
**Graduate**	0.010 [-0.087, 0.332]	0.019 [-0.060, 0.167]	0.013 [-0.062, 0.273]	0.003 [-0.114, 0.277]	-0.045 [-0.106, 0.451]
**<20K**	**0.023** [-0.079, 0.174]	-0.001 [-0.082, 0.141]	-0.036 [-0.085, 0.074]	-0.011 [-0.081, 0.111]	0.014 [-0.106, 0.115]
**20K–30K**	0.003 [-0.037, 0.103]	-0.007 [-0.065, 0.075]	-0.028 [-0.081, 0.043]	-0.018 [-0.080, 0.065]	0.009 [-0.068, 0.071]
**30K–50K**	-0.000 [-0.032, 0.069]	-0.007 [-0.032, 0.059]	-0.015 [-0.045, 0.105]	-0.009 [-0.083, 0.105]	0.004 [-0.056, 0.059]
**50K–100K**	-0.001 [-0.031, 0.149]	-0.002 [-0.020, 0.083]	0.004 [-0.013, 0.124]	0.001 [-0.017, 0.080]	0.004 [-0.025, 0.206]
**>100K**	-0.006 [-0.100, 0.410]	0.011 [-0.077, 0.149]	**0.029** [-0.060, 0.369]	0.018 [-0.080, 0.238]	-0.017 [-0.075, 0.645]

Note: All numbers come from the monthly average bias; outliers are included. The bold number is the maximum and minimum value of the column.

**Table 3:** Sampling biases of population groups at the county level from 2018 to 2022: Median [minimum, maximum] of all counties in the US. Most biases are in the range of [-0.056, 0.029].

Variable	Urban/Rural	2018	2019	2020	2021	2022
**Female**	Rural	0.002 [-0.168, 0.840]	0.002 [-0.188, 0.661]	0.001 [-0.137, 0.672]	0.002 [-0.108, 0.623]	0.002 [-0.109, 0.539]
	Urban	0.001 [-0.097, 0.366]	0.001 [-0.097, 0.438]	0.001 [-0.093, 0.464]	0.001 [-0.106, 0.480]	0.001 [-0.098, 0.474]
**Male**	Rural	-0.002 [-0.258, 0.129]	-0.002 [-0.256, 0.145]	-0.001 [-0.252, 0.106]	-0.002 [-0.243, 0.098]	-0.002 [-0.253, 0.089]
	Urban	-0.001 [-0.218, 0.119]	-0.001 [-0.246, 0.112]	-0.001 [-0.261, 0.103]	-0.001 [-0.270, 0.115]	-0.001 [-0.266, 0.107]
**15–17**	Rural	-0.009 [-0.568, 0.488]	-0.009 [-0.514, 0.699]	-0.010 [-0.460, 0.714]	-0.011 [-0.520, 0.683]	-0.011 [-0.552, 0.671]
	Urban	-0.010 [-0.231, 0.333]	-0.011 [-0.255, 0.912]	-0.003 [-0.406, 0.921]	-0.005 [-0.262, 4.317]	-0.007 [-0.240, 4.044]
**18–24**	Rural	-0.006 [-0.566, 0.357]	-0.009 [-0.552, 0.459]	-0.016 [-0.570, 0.656]	-0.012 [-0.503, 0.632]	-0.012 [-0.624, 0.965]
	Urban	-0.005 [-0.360, 0.662]	-0.012 [-0.278, 0.494]	-0.026 [-0.372, 0.555]	-0.019 [-0.332, 1.898]	-0.014 [-0.318, 1.872]
**25–34**	Rural	-0.007 [-0.626, 0.285]	-0.008 [-0.625, 0.375]	-0.010 [-0.644, 0.371]	-0.009 [-0.629, 0.398]	-0.009 [-0.586, 0.466]
	Urban	0.003 [-0.293, 0.185]	-0.003 [-0.306, 0.252]	-0.012 [-0.315, 0.186]	-0.013 [-0.327, 0.265]	-0.010 [-0.334, 0.243]
**35–44**	Rural	-0.006 [-0.551, 0.195]	-0.007 [-0.546, 0.263]	-0.007 [-0.559, 0.256]	-0.007 [-0.546, 0.319]	-0.008 [-0.512, 0.311]
	Urban	-0.001 [-0.327, 0.121]	-0.003 [-0.333, 0.150]	-0.001 [-0.364, 0.157]	-0.005 [-0.358, 0.173]	-0.005 [-0.345, 0.207]
**45–54**	Rural	0.001 [-0.308, 0.341]	0.000 [-0.281, 0.355]	0.003 [-0.219, 0.404]	0.000 [-0.288, 0.491]	0.001 [-0.357, 0.534]
	Urban	0.002 [-0.194, 0.192]	0.004 [-0.204, 0.218]	0.010 [-0.208, 0.171]	0.006 [-0.268, 0.279]	0.004 [-0.363, 0.311]
**55–64**	Rural	0.010 [-0.230, 0.449]	0.010 [-0.268, 0.515]	0.015 [-0.245, 0.421]	0.011 [-0.266, 0.416]	0.010 [-0.273, 0.378]
	Urban	0.007 [-0.193, 0.149]	0.011 [-0.140, 0.206]	0.017 [-0.121, 0.205]	0.015 [-0.210, 0.421]	0.014 [-0.264, 0.514]
**>65**	Rural	0.017 [-0.237, 0.820]	0.017 [-0.388, 0.642]	0.021 [-0.250, 0.647]	0.019 [-0.178, 0.594]	**0.017** [-0.239, 0.533]
	Urban	0.006 [-0.129, 0.277]	0.011 [-0.224, 0.603]	0.017 [-0.214, 0.618]	0.019 [-0.399, 0.819]	0.015 [-0.456, 1.029]
**White**	Rural	0.001 [-0.139, 0.507]	0.002 [-0.275, 0.591]	0.003 [-0.398, 0.813]	0.002 [-0.527, 1.055]	0.002 [-0.373, 0.867]
	Urban	0.000 [-0.152, 0.219]	0.003 [-0.142, 0.319]	0.006 [-0.083, 0.327]	0.005 [-0.158, 0.262]	0.005 [-0.120, 0.347]
**Black**	Rural	**-0.019** [-0.961, 1.629]	**-0.035** [-0.955, 2.344]	**-0.056** [-0.970, 2.099]	**-0.049** [-0.963, 2.478]	**-0.056** [-0.942, 2.914]
	Urban	0.000 [-0.731, 1.311]	-0.024 [-0.781, 2.970]	-0.052 [-0.763, 2.644]	-0.047 [-0.773, 3.857]	-0.043 [-0.769, 3.838]
**Hispanic**	Rural	-0.015 [-0.648, 1.294]	-0.026 [-0.720, 1.846]	-0.032 [-0.734, 1.223]	-0.027 [-0.728, 1.319]	-0.027 [-0.710, 2.349]
	Urban	-0.004 [-0.474, 0.487]	-0.020 [-0.666, 1.697]	-0.037 [-0.658, 0.567]	-0.034 [-0.674, 0.905]	-0.024 [-0.663, 1.106]
**Asian**	Rural	0.000 [-0.572, 3.246]	-0.010 [-0.713, 2.294]	-0.015 [-0.768, 4.057]	-0.013 [-0.801, 5.730]	-0.030 [-0.780, 4.716]
	Urban	0.018 [-0.724, 2.297]	0.003 [-0.691, 1.670]	-0.005 [-0.678, 1.344]	-0.008 [-0.689, 3.409]	-0.026 [-0.719, 2.063]
**NoSchool**	Rural	-0.010 [-0.426, 0.419]	-0.015 [-0.474, 0.327]	-0.017 [-0.460, 0.365]	-0.014 [-0.458, 0.392]	-0.009 [-0.492, 0.474]
	Urban	-0.008 [-0.451, 0.619]	-0.016 [-0.437, 0.564]	-0.025 [-0.437, 0.373]	-0.020 [-0.435, 0.433]	-0.007 [-0.429, 0.543]
**HighSchool**	Rural	0.000 [-0.346, 0.228]	0.000 [-0.367, 0.254]	0.002 [-0.357, 0.232]	0.002 [-0.345, 0.265]	0.003 [-0.357, 0.269]
	Urban	-0.000 [-0.251, 0.116]	-0.002 [-0.209, 0.168]	0.001 [-0.191, 0.208]	0.001 [-0.249, 0.251]	0.008 [-0.193, 0.342]
**NoCollege**	Rural	-0.002 [-0.341, 0.237]	-0.003 [-0.355, 0.163]	-0.002 [-0.346, 0.161]	-0.002 [-0.336, 0.199]	0.000 [-0.347, 0.215]
	Urban	-0.003 [-0.232, 0.140]	-0.006 [-0.213, 0.177]	-0.006 [-0.211, 0.209]	-0.003 [-0.272, 0.224]	0.004 [-0.217, 0.377]
**Bachelor**	Rural	0.020 [-0.288, 0.656]	0.020 [-0.438, 0.815]	0.023 [-0.493, 0.670]	0.019 [-0.465, 0.842]	0.013 [-0.470, 0.988]
	Urban	0.014 [-0.134, 0.341]	0.020 [-0.108, 0.351]	0.026 [-0.141, 0.363]	0.020 [-0.306, 0.386]	0.005 [-0.371, 0.421]
**Graduate**	Rural	**0.028** [-0.457, 0.874]	**0.026** [-0.302, 2.603]	**0.029** [-0.342, 1.670]	**0.024** [-0.304, 0.774]	0.013 [-0.356, 0.828]
	Urban	0.013 [-0.248, 0.653]	0.022 [-0.213, 0.699]	0.027 [-0.207, 0.895]	0.020 [-0.381, 1.062]	-0.001 [-0.456, 1.168]
**<20K**	Rural	0.002 [-0.460, 0.440]	-0.001 [-0.541, 0.459]	-0.007 [-0.518, 0.443]	-0.001 [-0.506, 0.419]	0.001 [-0.495, 0.447]
	Urban	0.009 [-0.171, 0.507]	-0.003 [-0.255, 0.580]	-0.022 [-0.287, 0.508]	-0.013 [-0.268, 0.602]	-0.003 [-0.267, 0.682]
**20K–30K**	Rural	0.000 [-0.343, 0.409]	0.000 [-0.598, 0.494]	-0.001 [-0.607, 0.567]	0.000 [-0.395, 0.725]	0.000 [-0.605, 0.883]
	Urban	0.002 [-0.391, 0.490]	-0.006 [-0.211, 0.367]	-0.017 [-0.223, 0.580]	-0.014 [-0.283, 0.418]	-0.005 [-0.249, 0.501]
**30K–50K**	Rural	-0.001 [-0.465, 0.262]	-0.001 [-0.292, 0.341]	-0.001 [-0.276, 0.317]	0.000 [-0.277, 0.597]	0.000 [-0.374, 0.865]
	Urban	0.002 [-0.177, 0.262]	-0.004 [-0.206, 0.358]	-0.009 [-0.204, 0.355]	-0.009 [-0.336, 0.322]	-0.001 [-0.274, 0.326]
**50K–100K**	Rural	-0.002 [-0.295, 0.361]	-0.002 [-0.268, 0.334]	0.000 [-0.239, 0.456]	-0.001 [-0.235, 0.483]	-0.002 [-0.223, 0.411]
	Urban	-0.003 [-0.179, 0.284]	-0.002 [-0.158, 0.375]	0.002 [-0.145, 0.398]	-0.001 [-0.224, 0.425]	0.001 [-0.211, 0.412]
**>100K**	Rural	0.002 [-0.360, 0.482]	0.005 [-0.249, 0.684]	0.008 [-0.301, 0.638]	0.001 [-0.350, 0.497]	-0.004 [-0.269, 0.621]
	Urban	0.001 [-0.424, 0.764]	0.009 [-0.361, 1.074]	0.022 [-0.361, 1.296]	0.015 [-0.395, 0.821]	0.001 [-0.386, 1.663]

Note: All numbers come from the monthly average bias; outliers are included. The bold number is the maximum and minimum value of the column.

From 2018 to 2022, the median bias of most population groups was relatively low, within the range of about ±0.05. At the state level, the data shows minor bias in gender over the five-year period (within ±0.0012). Population groups aged between 15–17, Hispanic, having no school, no college education, and income less than 50K are slightly underrepresented, with most bias values falling in [-0.059, 0.025]. The unrepresentativeness of the young groups is expected as SafeGraph does not track the mobile devices of children under the age of 16 [39]. In 2019, other age groups, Black, Asian, population with bachelor or graduate degrees, and income over 100K are generally overrepresented (about 0–0.02). It is noteworthy to mention that the population aged 65 and over is not generally underrepresented in SafeGraph data from 2020 to 2022. This maybe due to the gap between mobile phone and internet users and non-users no longer widens with age in the US [40], or this could be attributed to that this demographic group tends to consent to location-based cookie policies. The accessibility of internet infrastructure and the affordability of mobile phones have resulted in as many seniors using mobile phones as other age groups in the US. We also observed some unique patterns at the state level in 2022, where populations with lower education and income levels were overrepresented, while populations with higher education and income levels were underrepresented. This pattern contradicts the general understanding that mobile location data tends to underrepresent the population with low socioeconomic status.

In 2019, the overall bias for different population groups at the county level is generally consistent with the state level. The bias of race variables (i.e., Black, Hispanic, and Asian) exhibits the most significant variation among counties compared to other variables, while the bias of Male, Female, and White show the least variation with Female (median: 0.001–0.002) and White (median: 0–0.020) being slightly overrepresented. When examining the five-year period from 2018 to 2022, the bias for gender and all age groups shows consistent patterns, while other variables show notable changes and even change directions. For instance, Black and Asian groups were overrepresented in 2018 (median: 0–0.088), but their representativeness decreased in the following years, together with Hispanic.

The bias for most population groups exhibits urban/rural disparities over the five years, where the rural area generally shows less representation than the urban area, though with a few exceptions. The biases of these minority groups (i.e., Black, Asian) show a higher frequency of lower values in rural counties than in urban counties, and this pattern remained consistent over the five years, with the exception of Black populations in 2020 and 2021. For the Black and Hispanic populations, the rural population exhibited less representativeness than their urban counterparts in 2018. However, this gap reversed in the following 4 years.

#### 3.2.2 Spatial distribution of the demographic and socioeconomic bias

To examine the geographic disparities of the demographic and socioeconomic bias, we mapped the bias of each population group from 2018 to 2022 at both state and county levels. The maps for 2019 are shown in Figs 7 & 8, while the results for 2018, 2020, 2021, and 2022 are presented in Figs A26–A35 in S1 Appendix.

**Fig 7:**
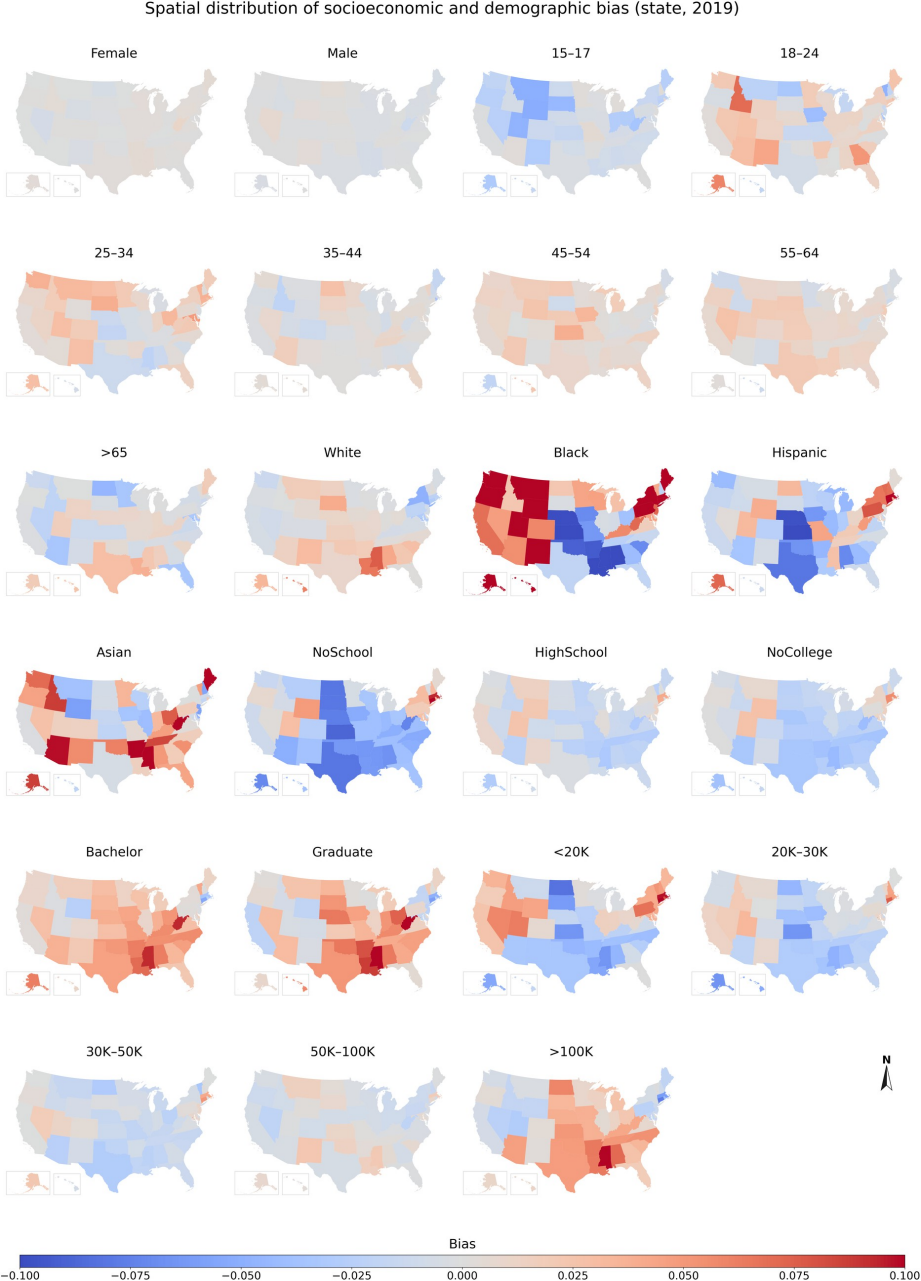
Spatial distribution of socioeconomic and demographic bias across population groups at state level in 2019. The results for 2018, 2020, 2021, and 2022 are presented in Figs A26, A28-A30 in S1 Appendix.

**Fig 8:**
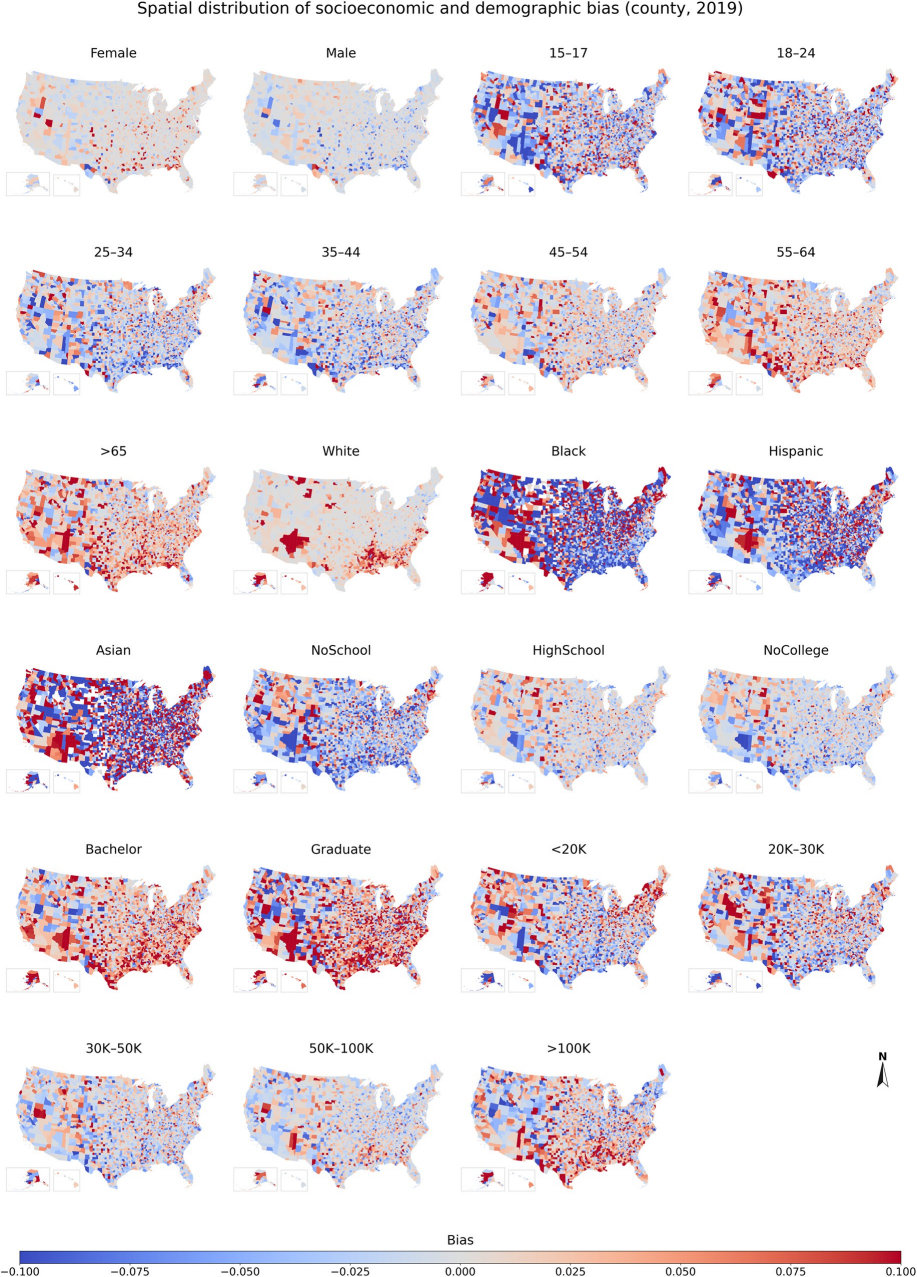
Spatial distribution of socioeconomic and demographic bias across population groups at county level in 2019. The results for 2018, 2020, 2021, and 2022 are presented in Figs A31, A33-A35 in S1 Appendix.

As shown in Fig 7, the data shows the bias in gender with no significant geographic disparities (light grey color) across the nation (within ±0.001). Bias in age exhibited marked geographic differences. The population group aged between 15–17 is underrepresented in most states (median: -0.009), with a relatively higher underrepresentation observed in the Northwest (e.g., Montana, North Dakota, and South Dakota). In contrast, population groups over the age of 17 were in general overrepresented in most states, with the highest overrepresentation in the West, the Northeast, Texas, and Florida in the South. These groups were only underrepresented in a few states, such as North Dakota (median: -0.043), Florida (median: -0.040), and Arizona (median: -0.036).

The bias in race/ethnicity varies across states. Among the four race groups, the data shows the best representativeness for the White, although there are slight overrepresentations in some Deep South states (i.e., Alabama, Georgia, Louisiana, Mississippi, South Carolina) and underrepresentations in certain Northeast (e.g., New York) and Western states (e.g., California). The bias in the Black is oppositely distributed from those of the White but generally shows overrepresentation. The Black was significantly overrepresented in the Northeastern and Western states and slightly underrepresented in some Central states (e.g., Nebraska, Kansas, and Alabama). The Hispanic was underrepresented in most states, particularly in some central states such as Kansas (-0.120), Nebraska (-0.110), and Texas (-0.090), while being overrepresented in the Northeast and Alaska (0.07). Asian was overrepresented in most states, with the highest overrepresentation in the South and West.

Population groups with no high school education were underrepresented in most states, especially in the Middel and South, and overrepresented in some states in Northeast (e.g., Connecticut, 0.088). Population groups with college education and over were overrepresented, particularly in the South. The maps also reveal that population groups with an income of 50K-100K have the best representativeness across all states. However, population groups with incomes below 50K were highly underrepresented in the South and Midwest, while being slightly overrepresented in some states in the Northeast and West. Meanwhile, population groups with incomes over 100K were generally overrepresented, particularly in the South and Midwest regions, with the highest overrepresentation observed in Mississippi (0.107) and Arkansas (0.068), though some states in the Northeast and West exhibited less representation.

To uncover more detailed geographic disparities, we further visualized the bias of different population groups at the county level (Fig 8). The results showed that the spatial distribution of the bias was generally consistent with those observed at the state level, except for Black and Asian groups. At the county level, the underrepresented and overrepresented counties for Black/Asian were relatively dispersed, with no clear clustering trend (except in the Southwest, where Black/Asian is overrepresented). However, at the state level, the Black and Asian were overrepresented in many states, particularly in the West and Northeast.

The geographic disparities of other years show consistency with those of 2019 at both county and state levels, albeit with some variations at the state level. For instance, the Black and Asian were overrepresented in most states in 2018 and 2019, while they were underrepresented in most states in 2020, 2021, and 2022. Specifically, from 2020 to 2022, the Black was underrepresented in almost all Midwestern states, and the Asian was underrepresented in a growing number of states. In 2018, Hispanics were only underrepresented in some southern states, such as Nebraska (-0.078), Kansas (-0.066), Alabama (-0.048), and Texas (-0.046). Moreover, the pandemic exacerbated geographic disparities in the sampling bias for different population groups. In 2020 and 2021, minority and low-income groups were highly underrepresented in more states, while the White and high-income groups were highly overrepresented in more states.

Overall, the spatial distribution of the bias at the state and county levels across the five years shows that youth, minorities, low-income groups, and those with lower levels of education were more likely to be underrepresented in the South and Midwest, while their counterparts (i.e., the White, groups with higher income and higher education levels) were more likely to be overrepresented in the South. Moreover, we found that the geographic disparities of the bias vary across the years, with the pandemic exacerbating geographic disparities in the sampling bias for different population groups.

#### 3.2.3 Temporal trend of the demographic and socioeconomic bias

**Fig 9:**
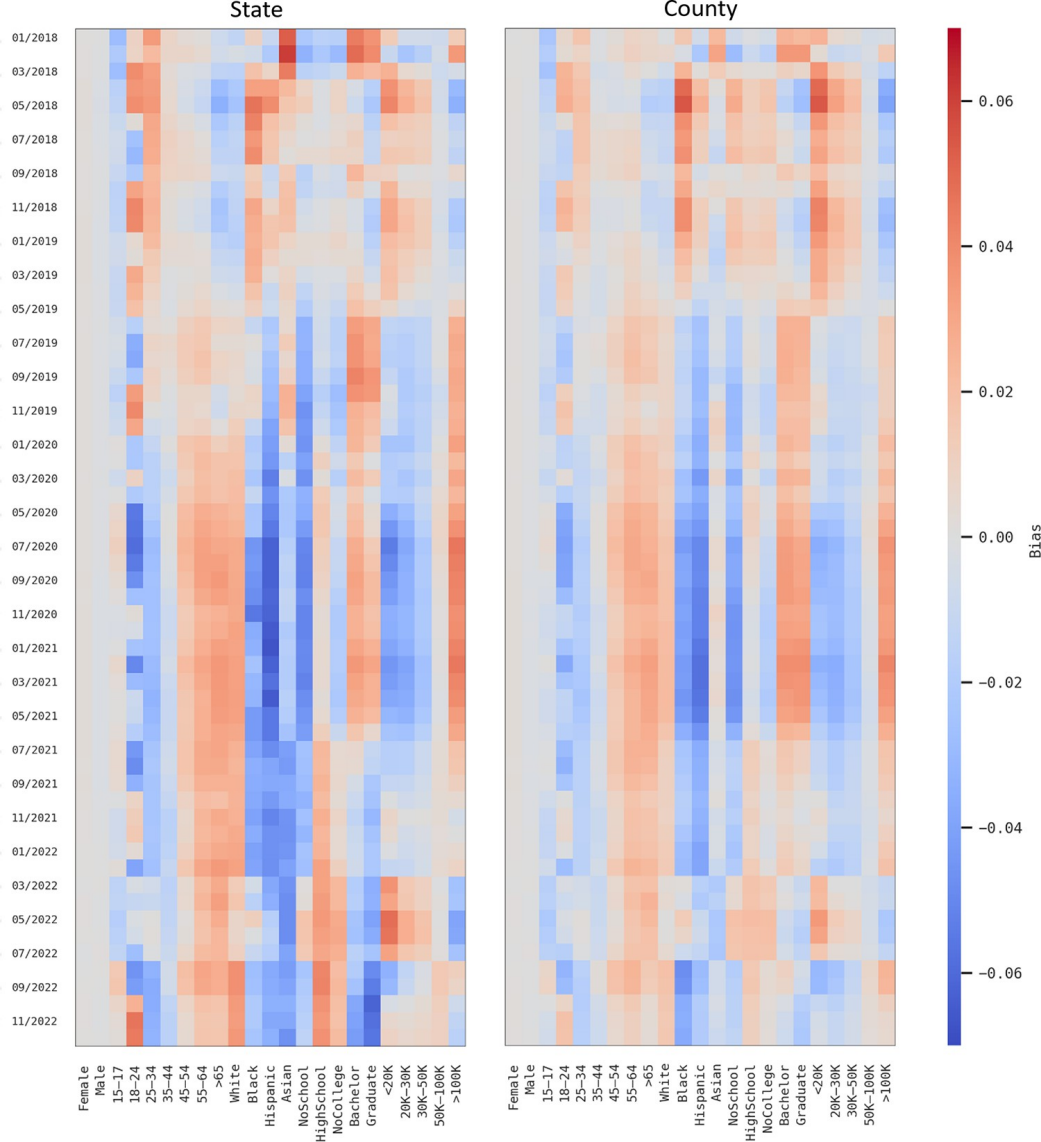
Monthly trend of the socioeconomic and demographic bias across population groups at county and state levels from 2018 to 2022.

Besides the observed geographic disparities, the demographic and socioeconomic bias shows noticeable changes over the five years as illustrated in sections 3.2.1 and 3.2.2. In this section, we further analyzed the monthly trend of the bias of each population group from 2018 to 2022 for both county and state levels using heatmaps (Fig 9).

At both county and state levels, the temporal trend for the two geographic levels is similar throughout the five years. The monthly bias across groups shows similar patterns as the overall bias (Figs A21-A25 in S1 Appendix). The gender shows minimal bias and is stable over time. Adult groups (those aged 18 and over) were slightly of oversampled (0.01–0.03), and those aged under 18 were underrepresented marginally. The patterns are in general consistent over time. Among racial groups, Hispanics were slightly overrepresented in 2018 and early 2019. However, their representation gradually decreased, with a significant underrepresentation (-0.05 –-0.02) observed following the pandemic (March 2020) to July 2021. Asians and Whites are generally overrepresented with an inconsiderate monthly bias of 0.01. Those with a bachelor’s degree or higher were overrepresented (0.03) than others with lower education levels (-0.02). Similarly, the high-income group (>100K) displays an opposite trend (0.01–0.03), but the low-income population (<50K) shows a minor underrepresentation. The median income households (50K –100K) were well sampled. The monthly bias among various income groups was minor (-0.02–0.02). It is important to note that this negligible bias resulted from the average of 3,220 counties or 52 states (weighted by population); the bias fluctuates among counties or states with a large variance (see the violin plots in Fig 6).

Understanding the temporal changes in demographic and socioeconomic bias is crucial. While some population groups exhibit a consistent pattern of either overrepresentation or underrepresentation, the bias of some groups varies across months. During the pandemic, individuals from low socioeconomic groups (including Black and Hispanic individuals, those with less than a college education, and those with a household income below $50K) were found to be significantly underrepresented compared to other periods as demonstrated by the changing to darker blue colors following the pandemic. This finding suggests that the COVID-19 outbreak may foster disparities in the sampling representation of vulnerable groups. This is not unexpected, as the pandemic has highlighted digital inequalities that are well-known in public health research [41, 42].

## 4. Limitations and future research

We conducted a comprehensive bias analysis on the emerging mobile location dataset. Although this effort represents one of the first attempts in the literature, there are some limitations to this approach. First, our bias detection approach relies on aggregation-based methods [28, 29], and such methods are not adequate for detecting biases at fine-grained levels, such as census tracts and block groups with limited sampled devices (dozens). We advocate novel methods to detect the sampling bias among population groups at those levels.

Second, our analysis is limited in its coverage of other components of the mobile location data. Specifically, this study focused on the sampled device count; other important components of the SafeGraph monthly Patterns dataset, such as sampled POIs and visit counts at the sampled POIs, were not fully addressed. One potential avenue for future research could involve using high schools with known student counts to examine the bias of sampled high school POIs and visit counts. Furthermore, it should be noted that SafeGraph has added Laplacian noise to critical columns in Patterns, such as visitor home CBG, for privacy reasons [31]. Following the addition of this noise, the SafeGraph dataset only featured census block groups (CBGs) with a minimum of two devices. In instances of 2–4 devices, the count was marked as four. While this noise addition is generally considered to minimally affect large-scale, aggregated data, its influence on smaller areas like CBGs is still uncertain. Consequently, the specific impact of the added noise on any data biases requires further investigation.

The third limitation of this study is the inconsistency in boundary data between the Census Bureau and SafeGraph. While the Bureau implemented updated geographical boundaries in 2020, SafeGraph continued to use the 2010 boundaries. For the sake of consistency, this study utilized the Census Bureau’s 2019 boundaries and the associated ACS 2019 data. Census Bureau provides CBG level crosswalk files to compare two Census results [35]. We noticed that there are about 173,585 Census block groups (CBGs) out of 220,333 block groups in Census 2010 that have the same GEOID in 242,335 CBGs in Census 2020. The extent to which these changes in boundaries have influenced the findings of this study remains unclear since this issue was not examined in the current paper.

Finally, we used the ACS 2019 5-year estimation in analyzing the bias for the 2020–2022 SafeGraph datasets. While we believe that the marginal population change (1.01% nationwide increase from 2019 to 2022) [32] will not significantly impact our conclusions, it is possible that these numbers may not be entirely accurate. Therefore, caution should be exercised when interpreting the results of our study, particularly with respect to population-level estimates. Further research and analysis are needed to refine our understanding of the impact of these factors on bias in mobile location data.

## 5. Conclusion

The use of mobile location data for investigating human mobility patterns has become increasingly important in various research domains, and SafeGraph is one of the most commonly used sources of such data. In this study, we comprehensively examined the sampling bias of SafeGraph Patterns across five dimensions of spatial, temporal, urbanization, demographic, and socioeconomic, covering the entire US over the past five years from 2018 to 2022. While our analysis focused on SafeGraph data in the US, the analysis approach detailed in this study can be readily applied to other mobile location data such as Advan Patterns [6], other geographic regions (e.g., Canada and Europe), and other time periods.

The SafeGraph Patterns dataset exhibited a fluctuating sampling rate over the past five years, with an average of 7.5%, which is relatively large given the size of the US population. The sampling rate was relatively uniform at the county level, and the number of sampled devices was strongly correlated with the census population for both urban (*r* > 0.97) and rural counties (*r* > 0.91), but less so at the tract and CBG levels. The sampling bias was generally minor among population groups such as gender, age, and moderate-income, with biases typically falling within the range of -0.05 and +0.05. However, minority groups such as Hispanic populations, low-income households, and individuals with low levels of education generally exhibited higher levels of underrepresentation bias that varied over space, time, urbanization, and across spatial scales. We also observed a notable increase in the underrepresentation of low socioeconomic groups following the COVID-19 pandemic, indicating that the COVID-19 outbreak may exacerbate pre-existing disparities in the representation of vulnerable groups within the data. These findings provide important insights for future studies that utilize SafeGraph data or other mobile location datasets, highlighting the need to thoroughly evaluate the spatiotemporal dynamics of the bias across spatial scales when employing such data sources.

To ensure the accuracy and validity of results when analzying mobile location data, we recommend that future studies using such data sources carefully consider the sampling biases from multiple dimensions and employ appropriate approaches to mitigate these biases. These approaches may include applying statistical weighting to adjust the data to reflect the true distribution of the population of interest, conducting sensitivity analyses to assess the impact of sampling bias on the results, and combining with other data sources, such as social media and census data, to provide additional information about the characteristics of the population.

## Supporting information

S1 Appendix This appendix includes the results of the bias analysis for the five years from 2018 to 2022. Note that figures for 2019 are also included to facilitate comparison with other years.
(DOCX)
