# Supplementary material for: Understanding the bias of mobile location data across spatial scales and over time: A comprehensive analysis of SafeGraph data in the United States
Source: PLoS One. 2024 Jan 19;19(1):e0294430. doi: 10.1371/journal.pone.0294430 (PMC10798630; doi:10.1371/journal.pone.0294430)
Supplement: S1 Appendix — Note that figures for 2019 are also included to facilitate comparison with other years. (DOCX) [file pone.0294430.s001.docx]

**Appendix**

This appendix includes the results of the bias analysis for the five years from 2018 to 2022. Note that figures for 2019 are also included to facilitate comparison with other years.


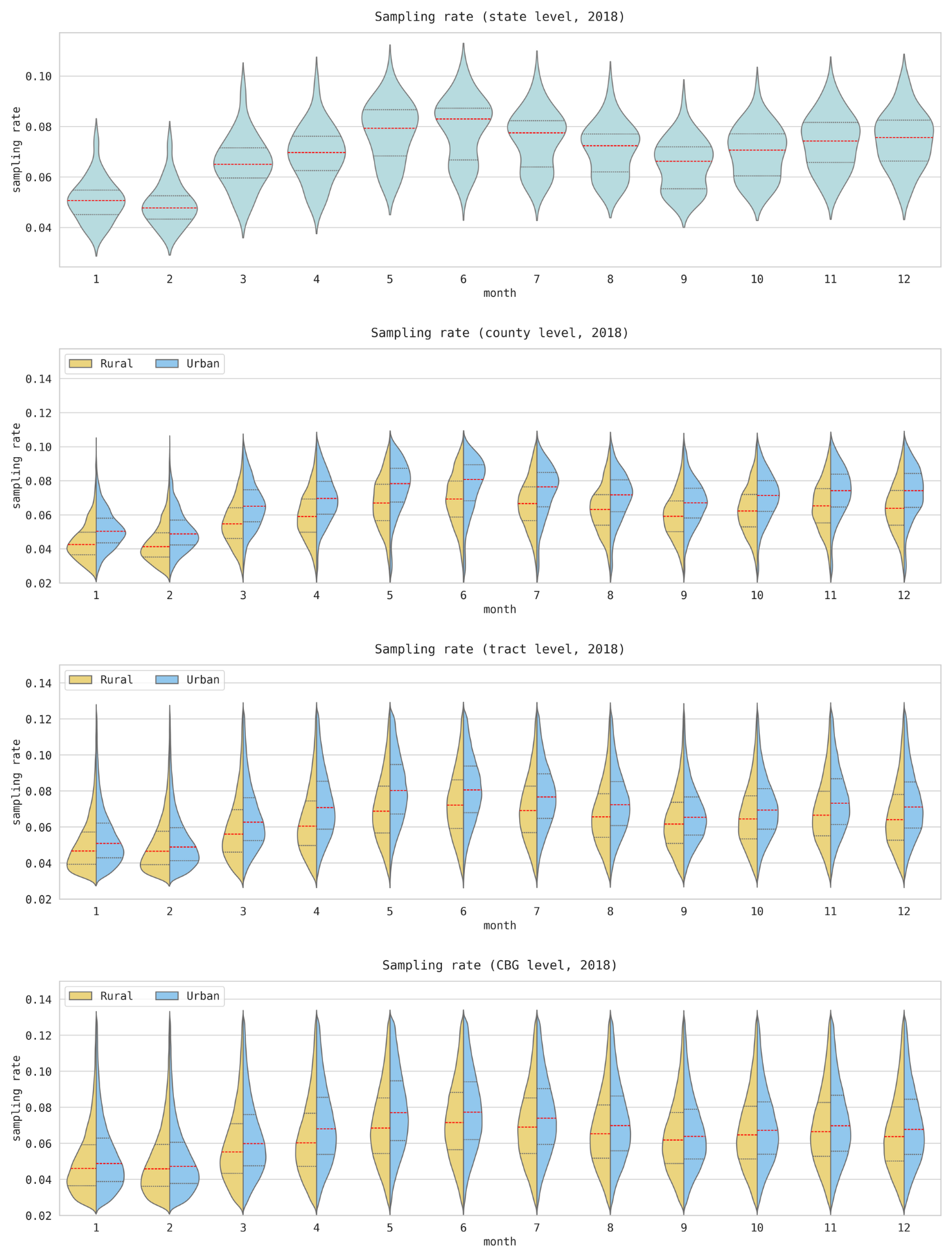


**Figure A1. Distribution of the monthly sampling rate at the four geographic levels in 2018**


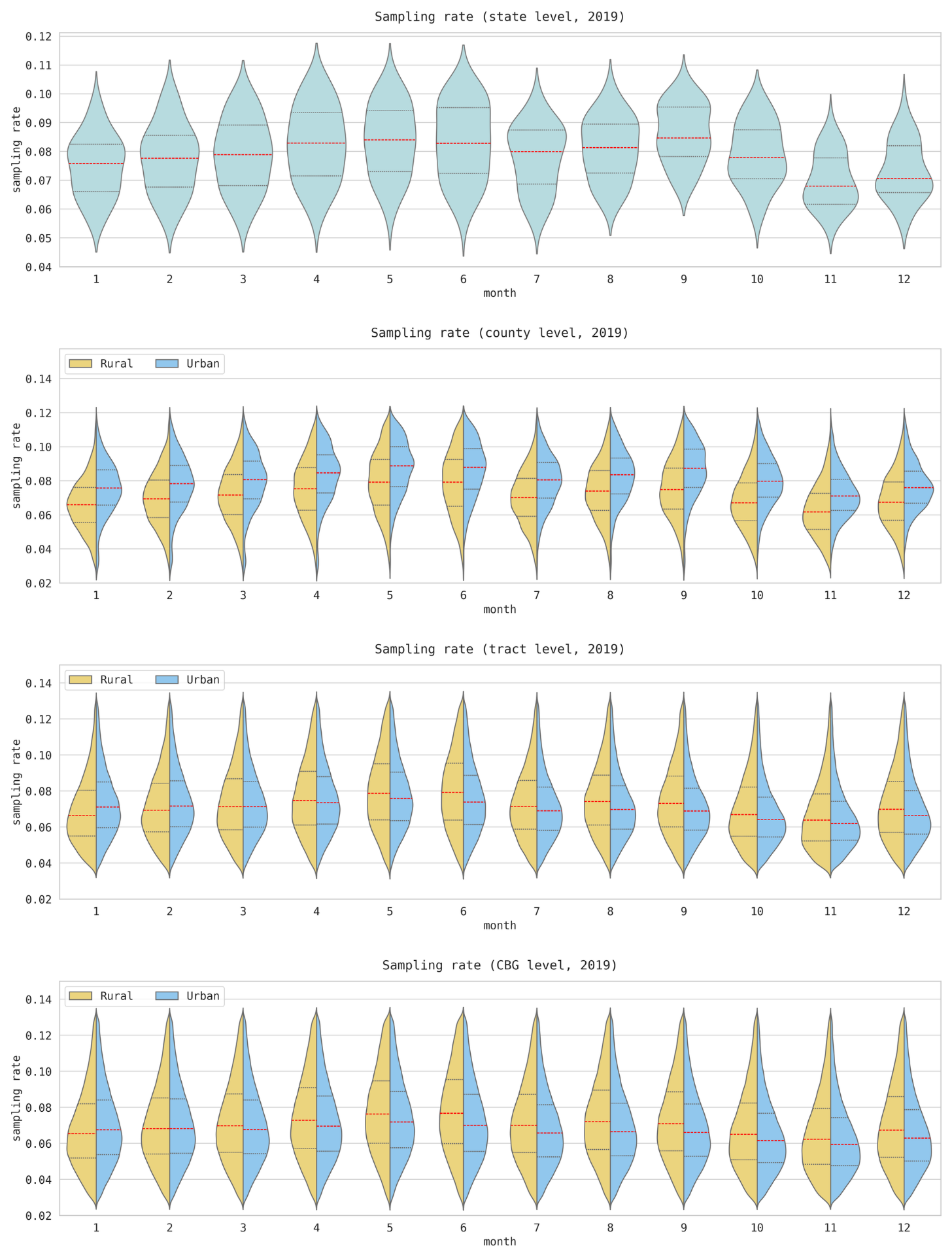


**Figure A2. Distribution of the monthly sampling rate at the four geographic levels in 2019**


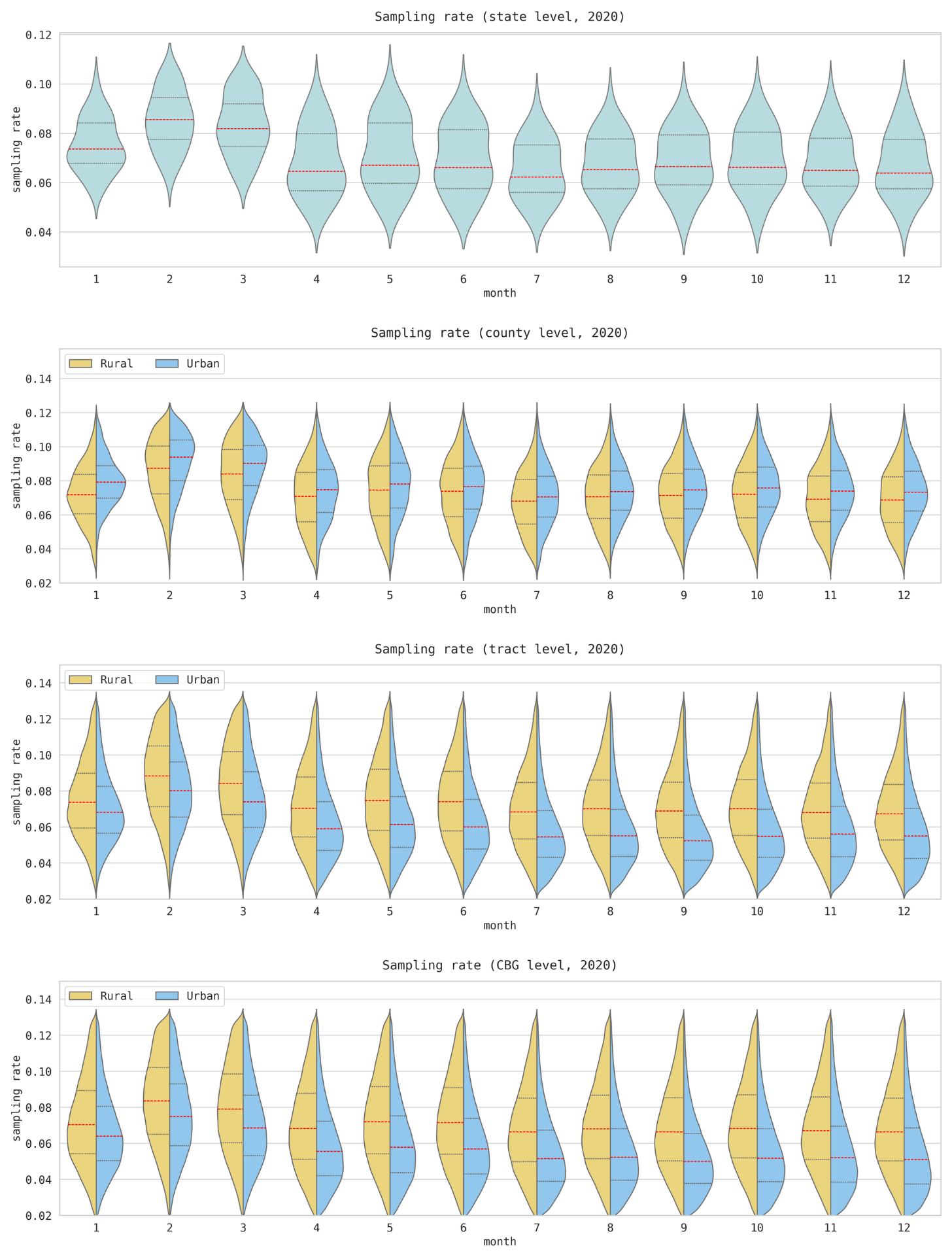


**Figure A3. Distribution of the monthly sampling rate at the four geographic levels in 2020**


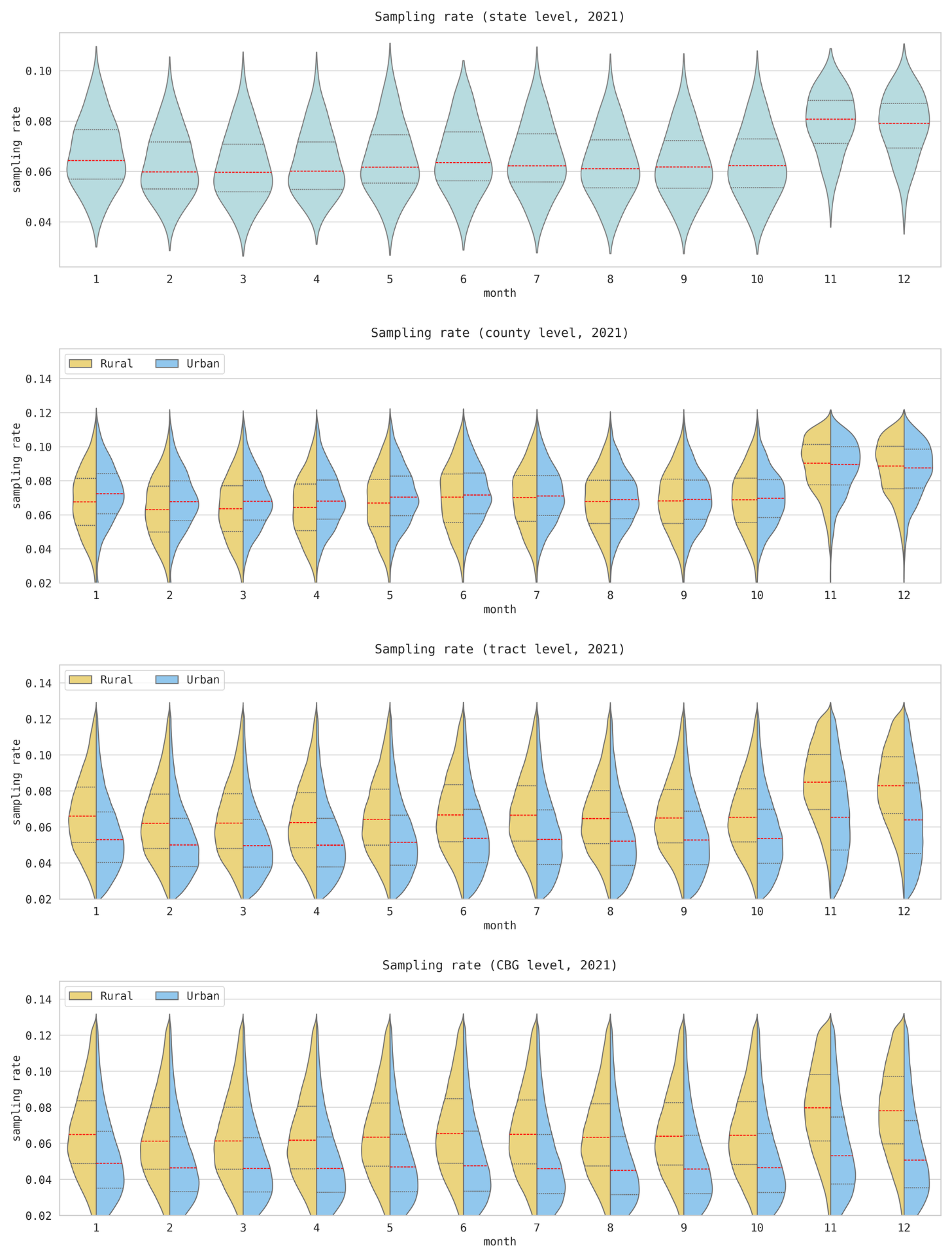


**Figure A4. Distribution of the monthly sampling rate at the four geographic levels in 2021**


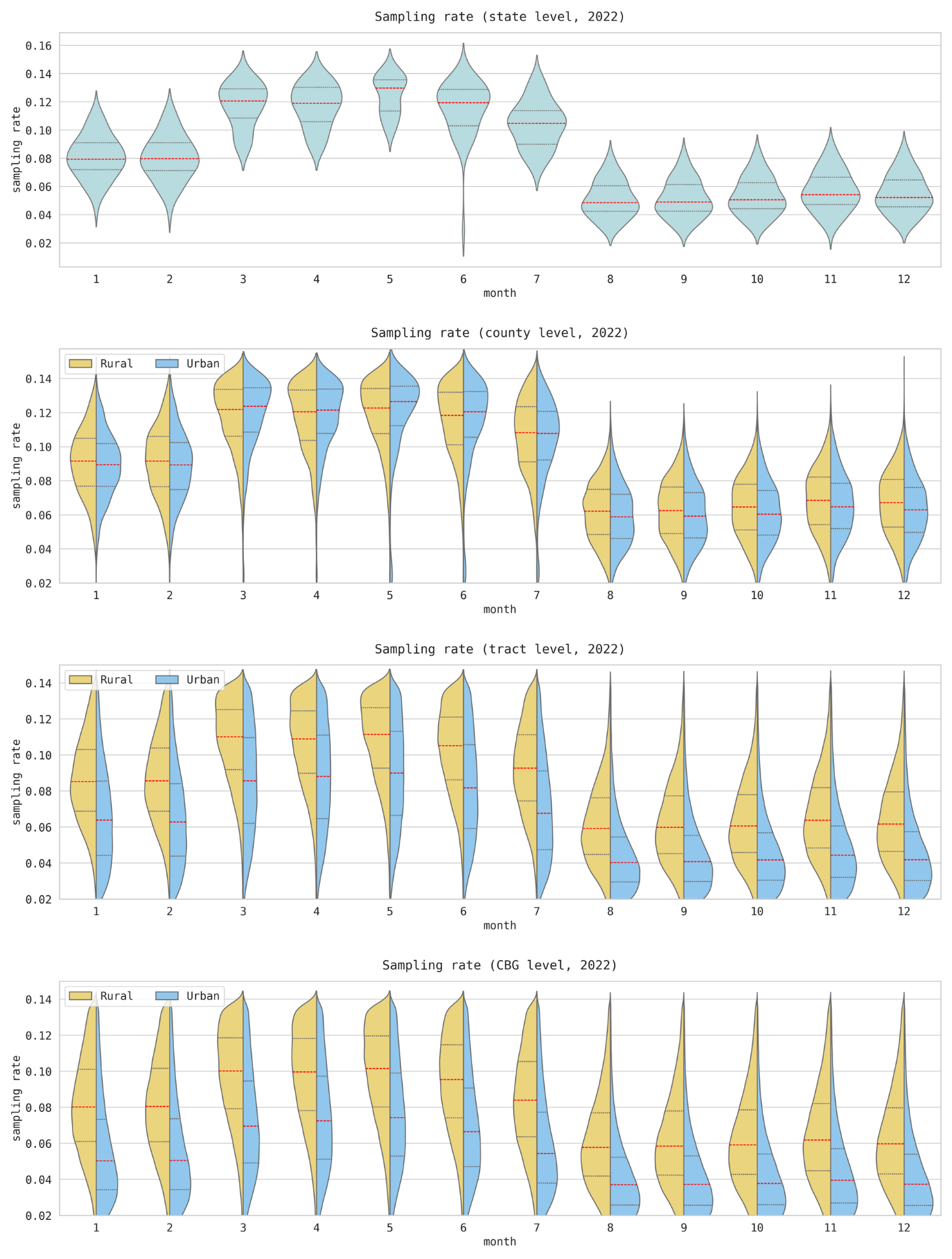


**Figure A5. Distribution of the monthly sampling rate at the four geographic levels in 2022**


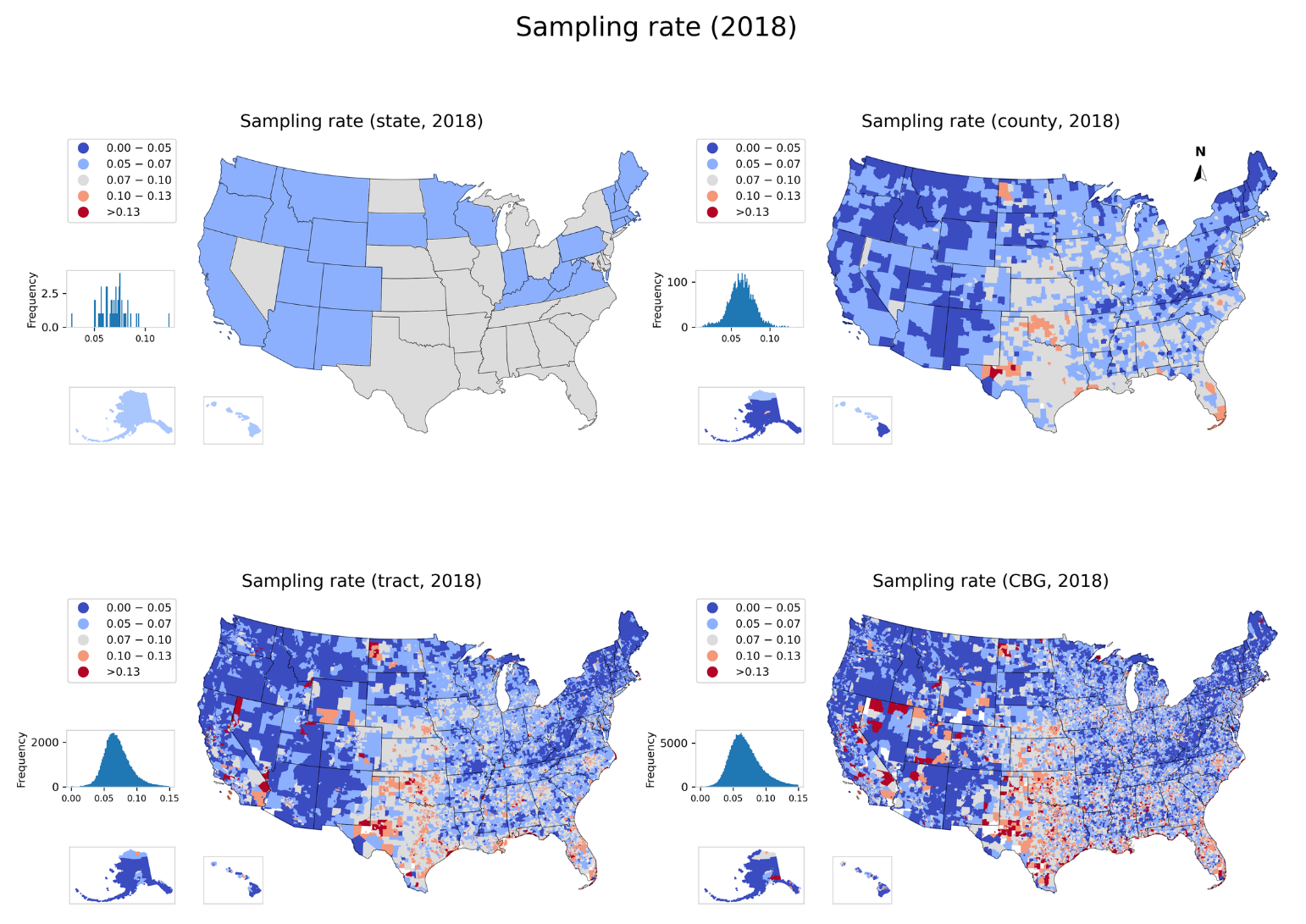


**Figure A6. Spatial distribution of the sampling rate in 2018 across four geographic levels**


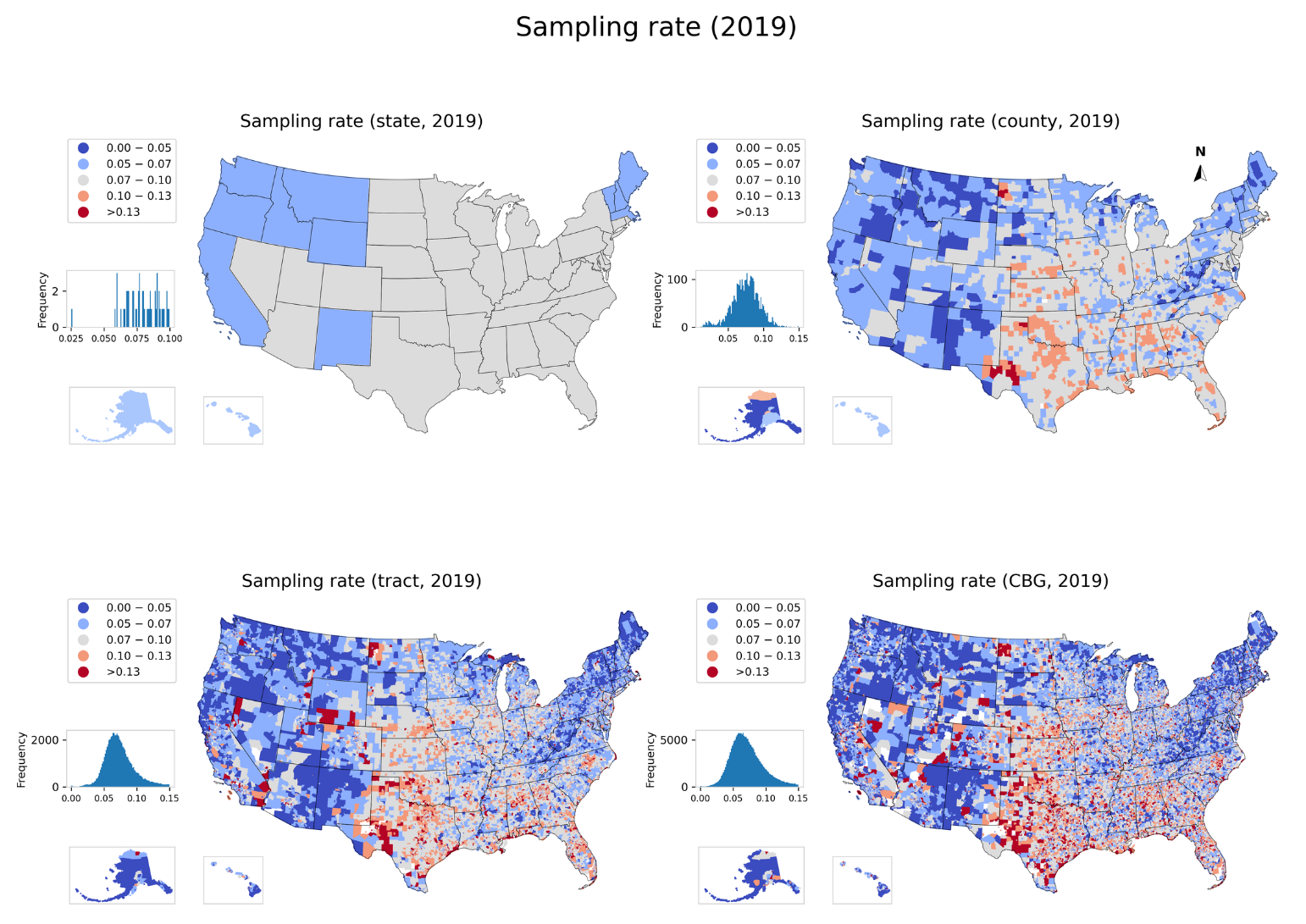


**Figure A7. Spatial distribution of the sampling rate in 2019 across four geographic levels**


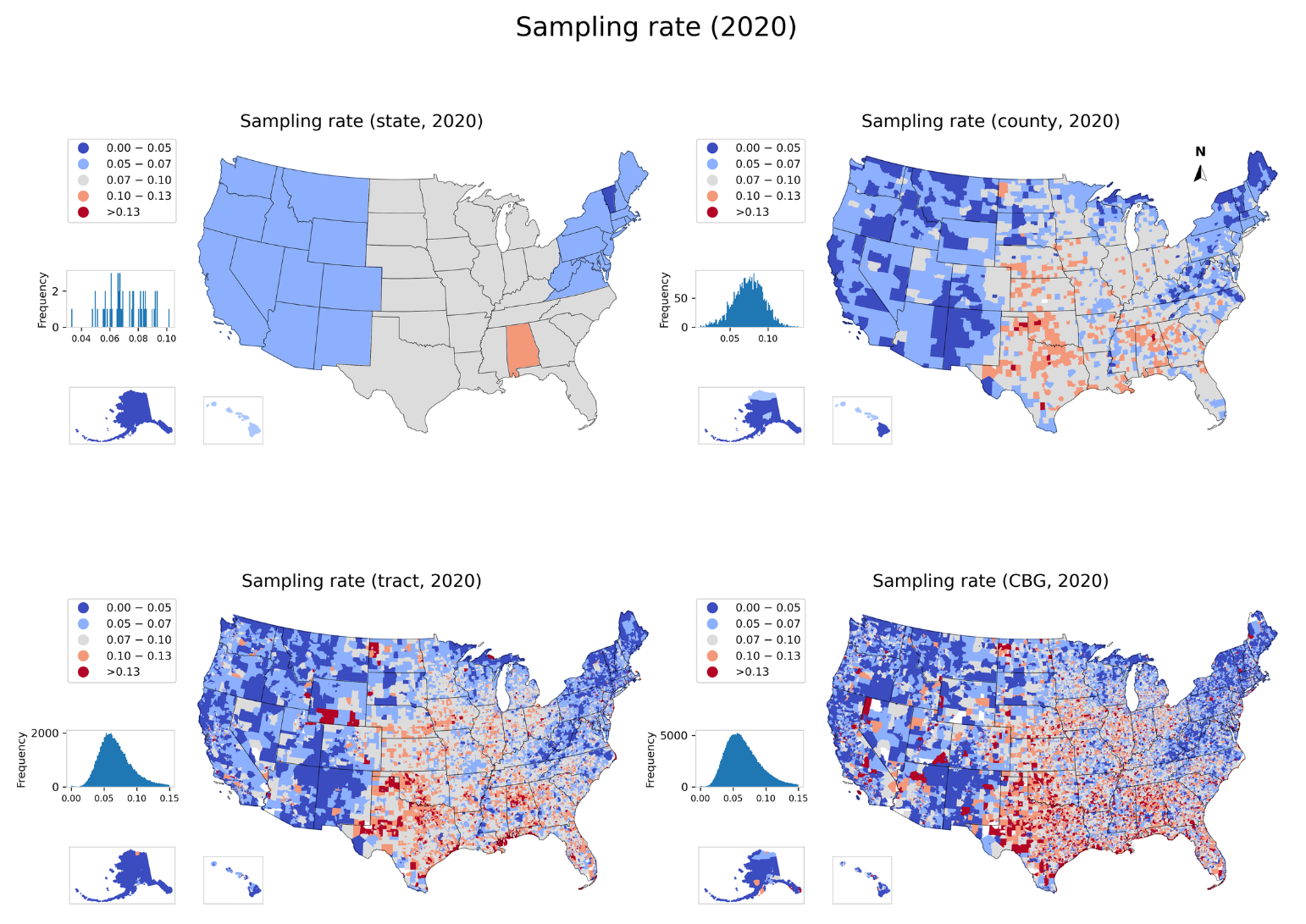


**Figure A8. Spatial distribution of the sampling rate in 2020 across four geographic levels**


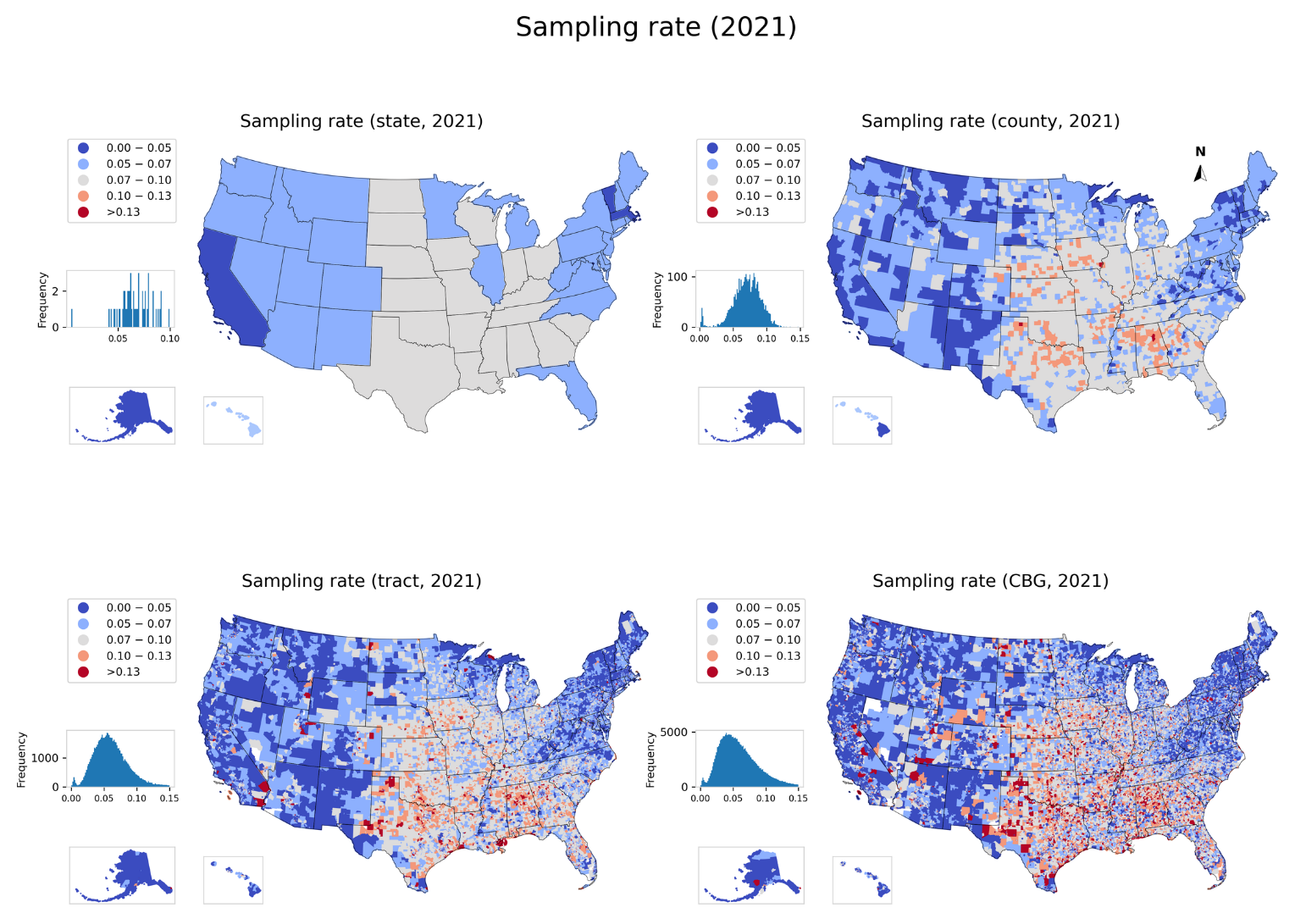


**Figure A9. Spatial distribution of the sampling rate in 2021 across four geographic levels**


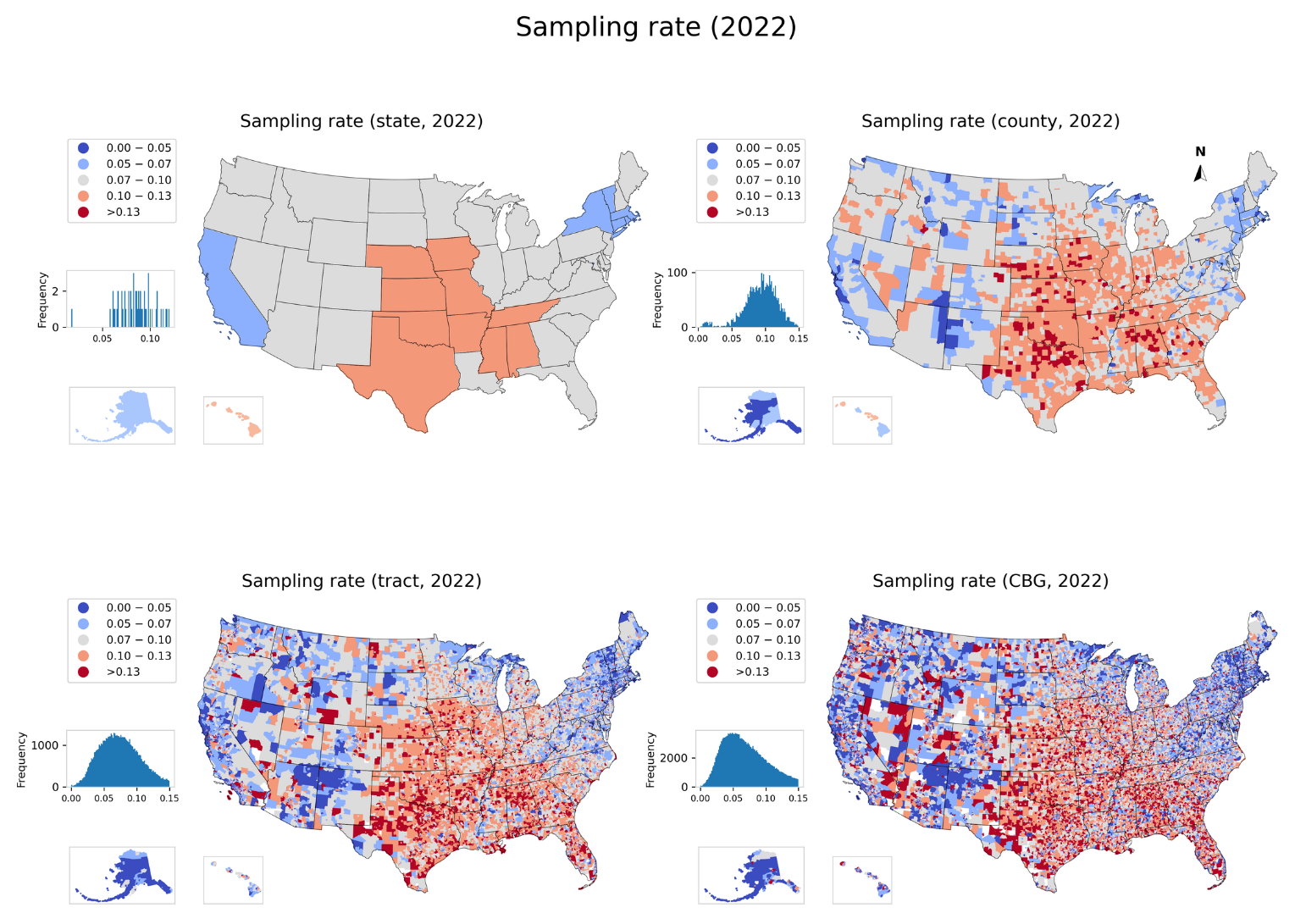


**Figure A10. Spatial distribution of the sampling rate in 2022 across four geographic levels**


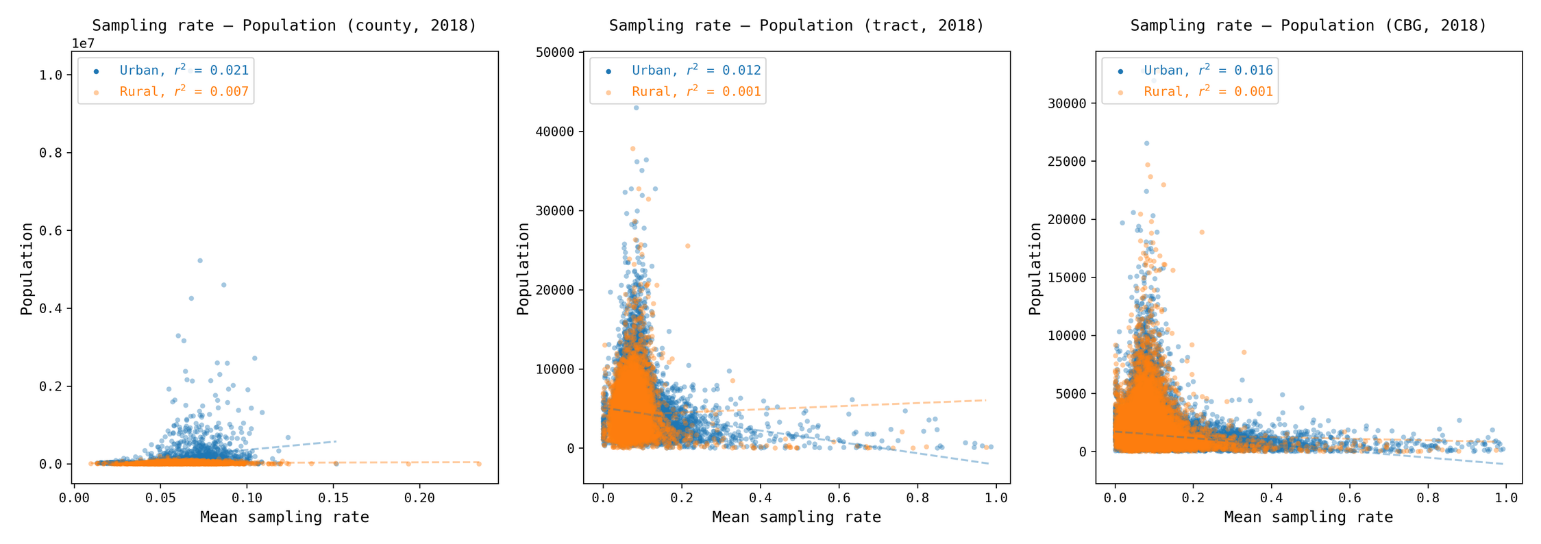


**Figure A11. The correlation between the sampling rate and the census population for 2018**


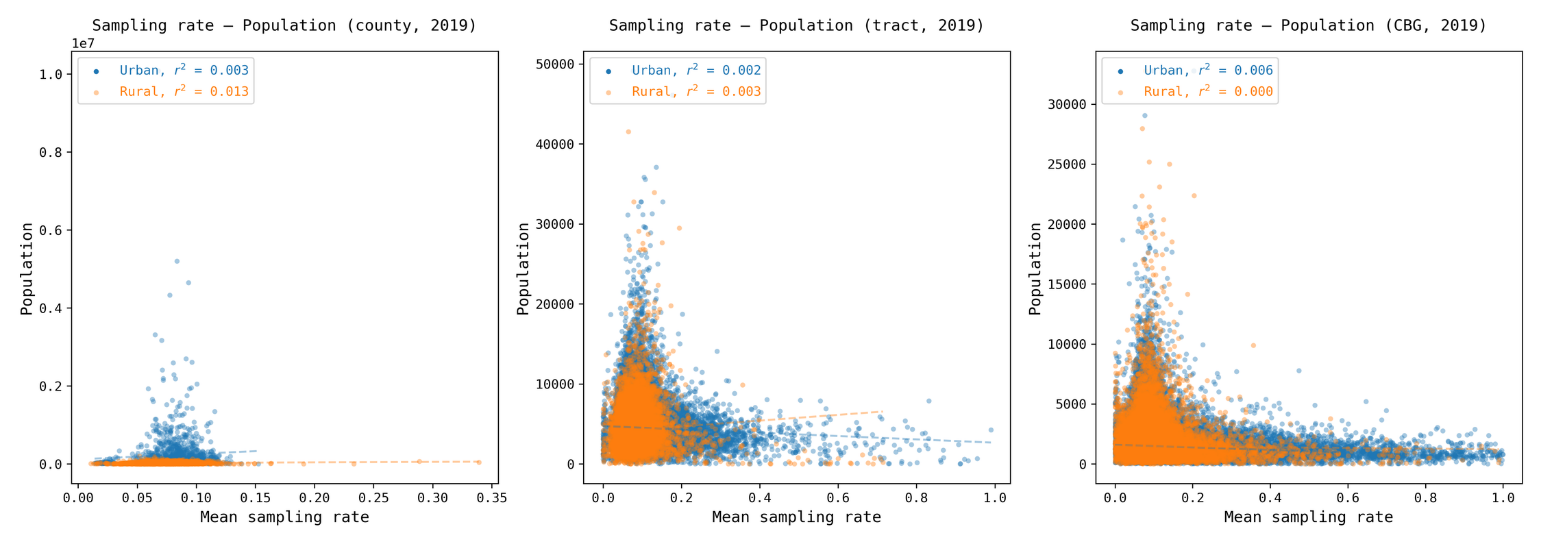


**Figure A12. The correlation between the sampling rate and the census population for 2019**


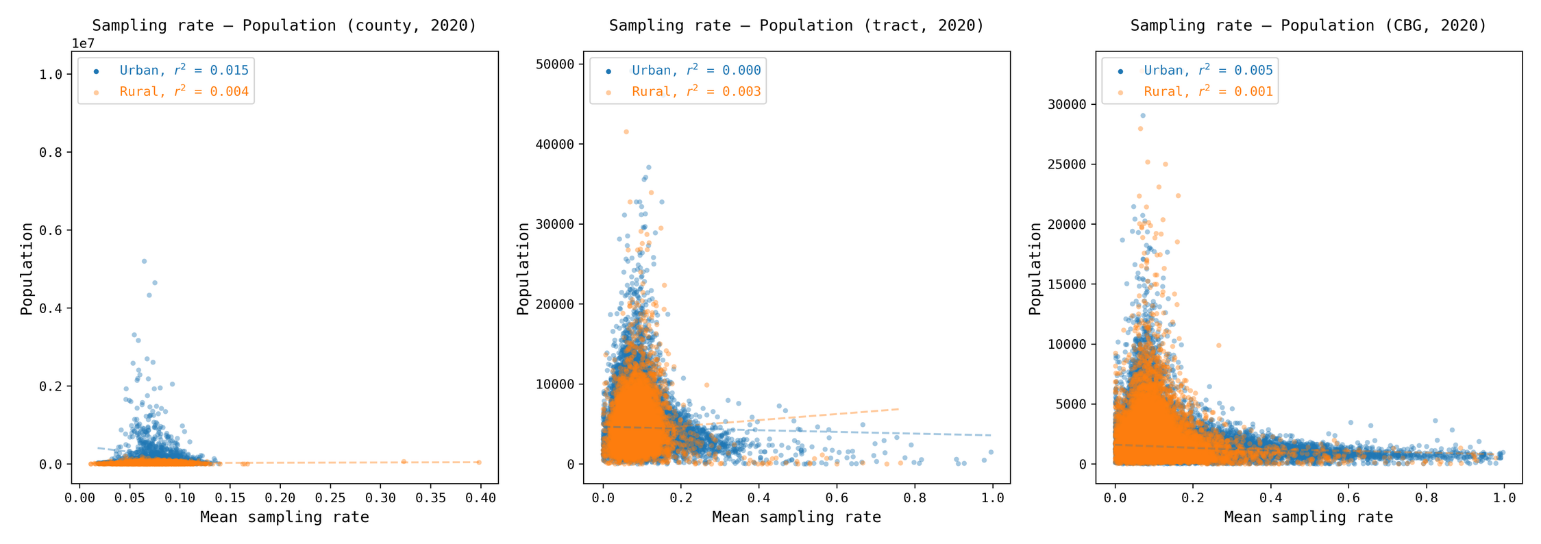


**Figure A13. The correlation between the sampling rate and the census population for 2020**


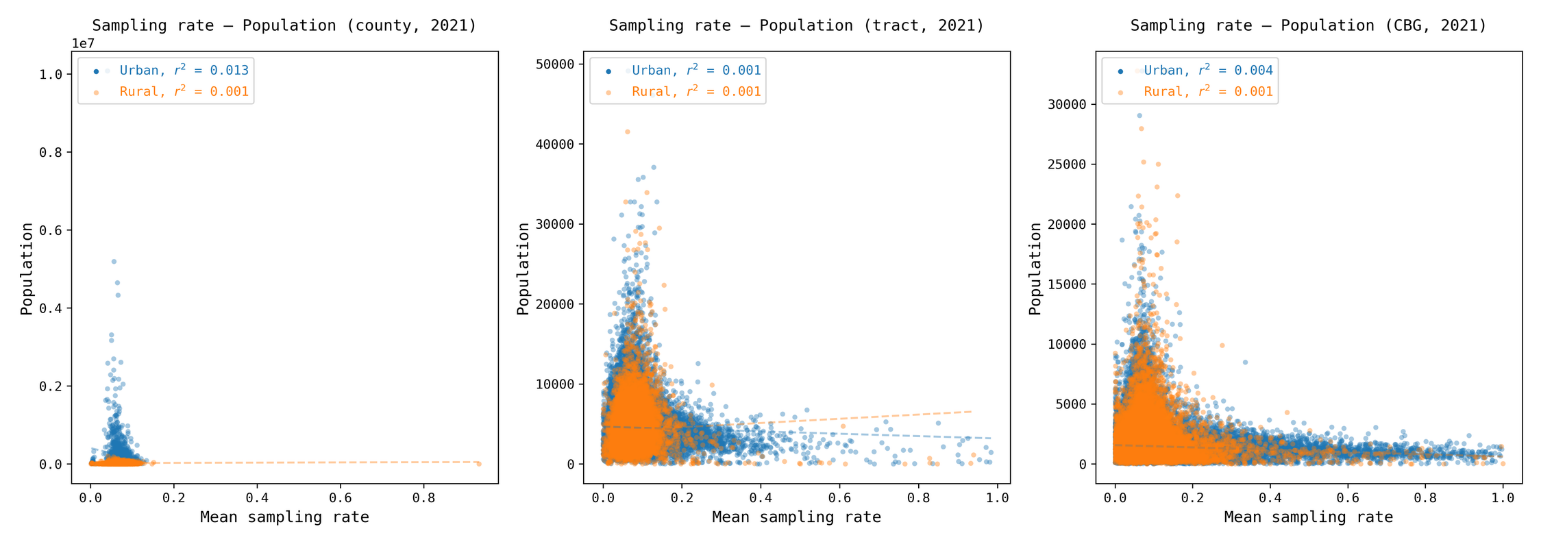


**Figure A14. The correlation between the sampling rate and the census population for 2021**


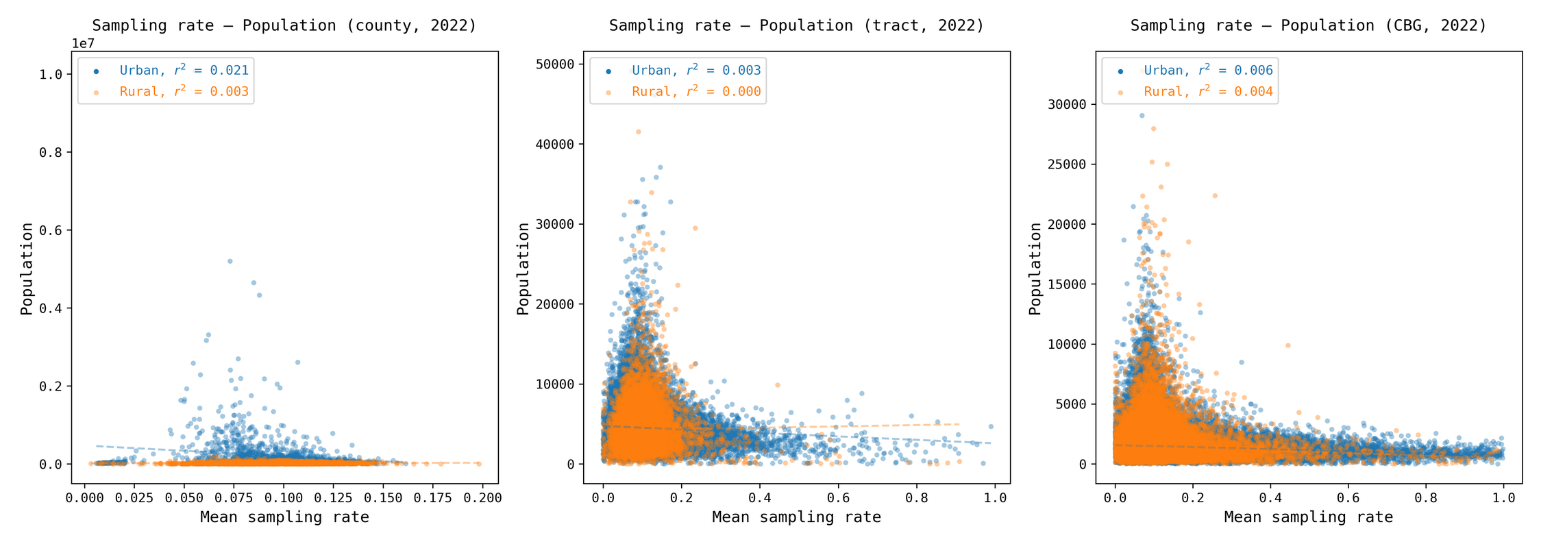


**Figure A15. The correlation between the sampling rate and the census population for 2022**


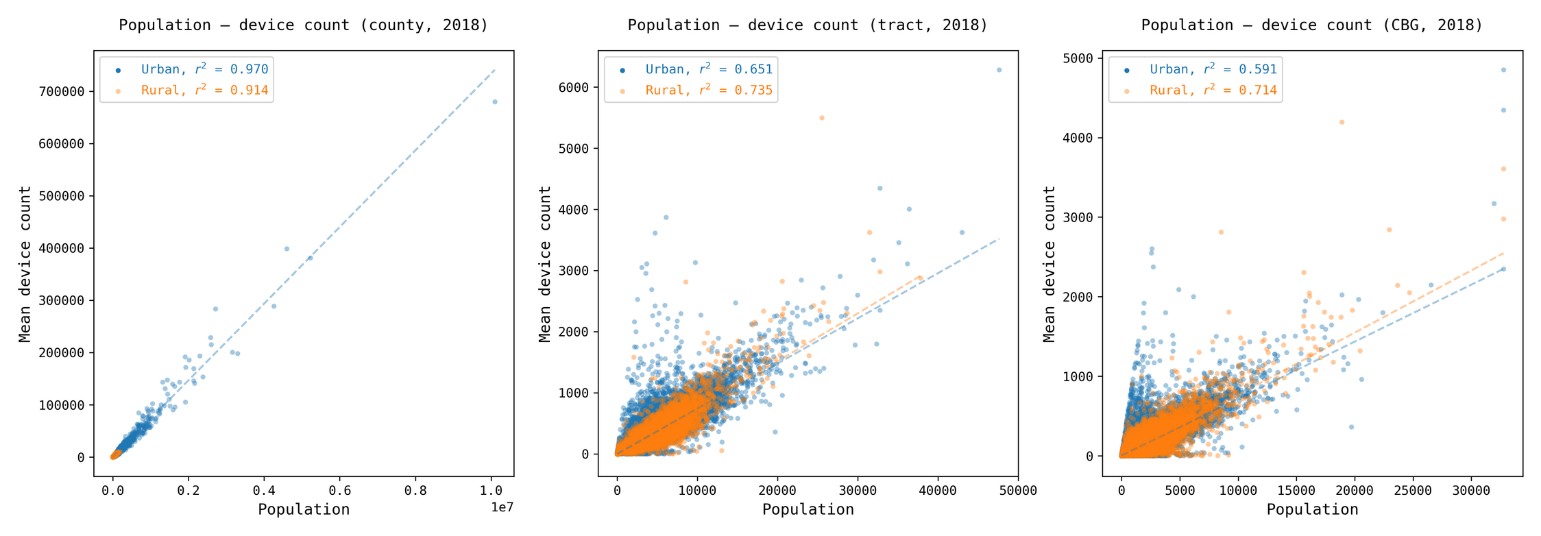


**Figure A16. Correlation between device count and census population for 2018**


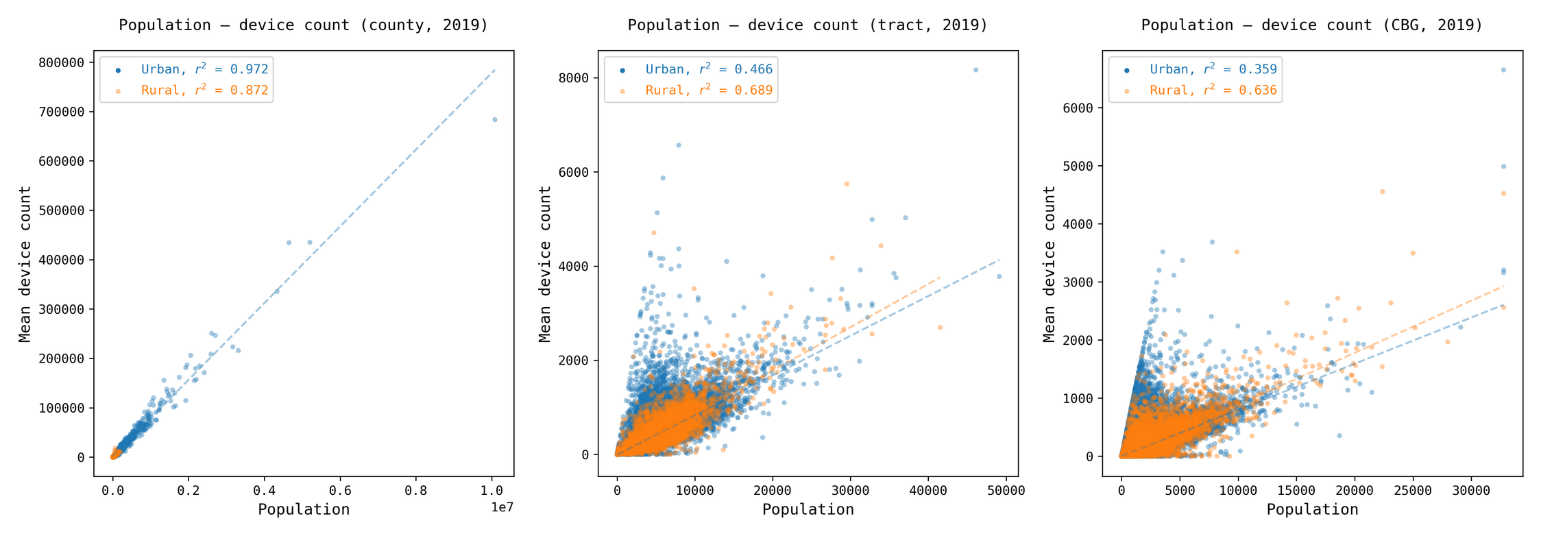


**Figure A17. Correlation between device count and census population for 2019**


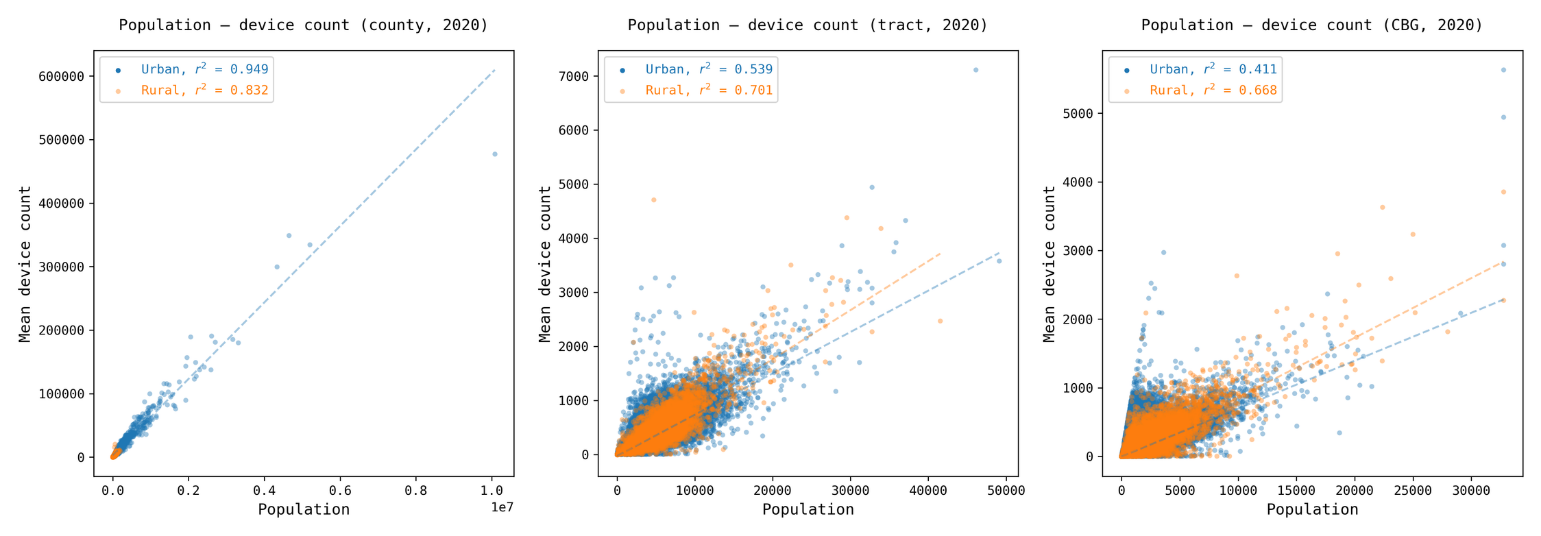


**Figure A18. Correlation between device count and census population for 2020**


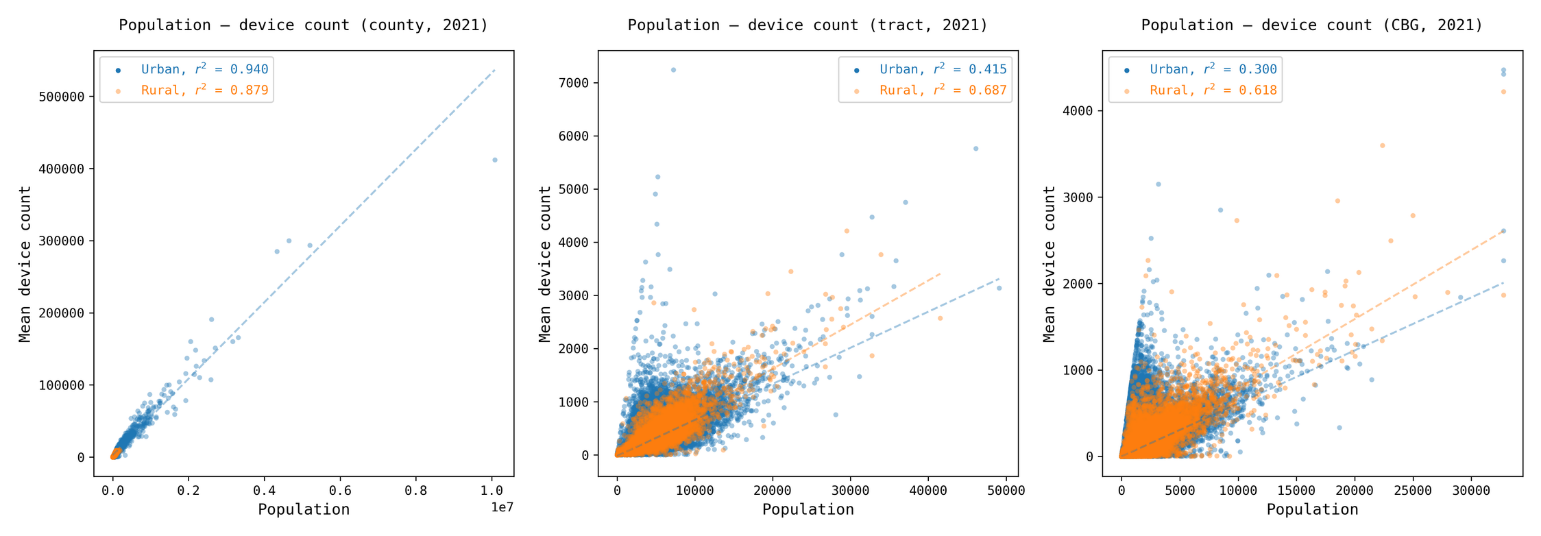


**Figure A19. Correlation between device count and census population for 2021**


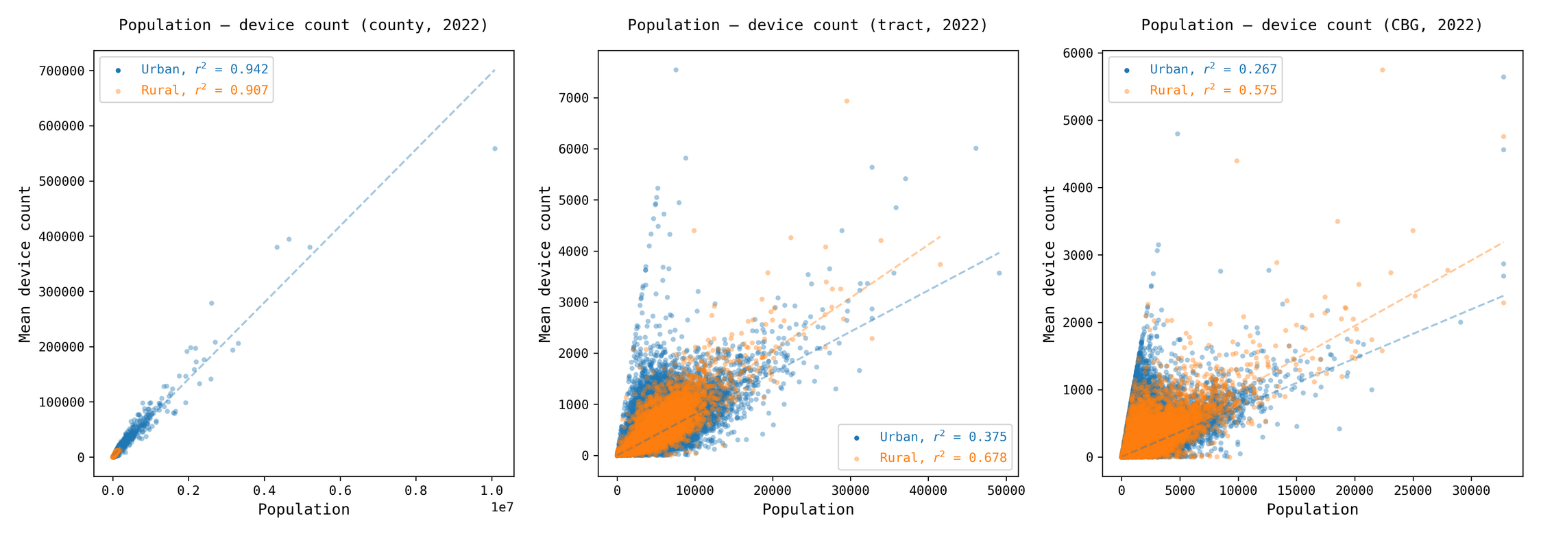


**Figure A20. Correlation between device count and census population for 2022**


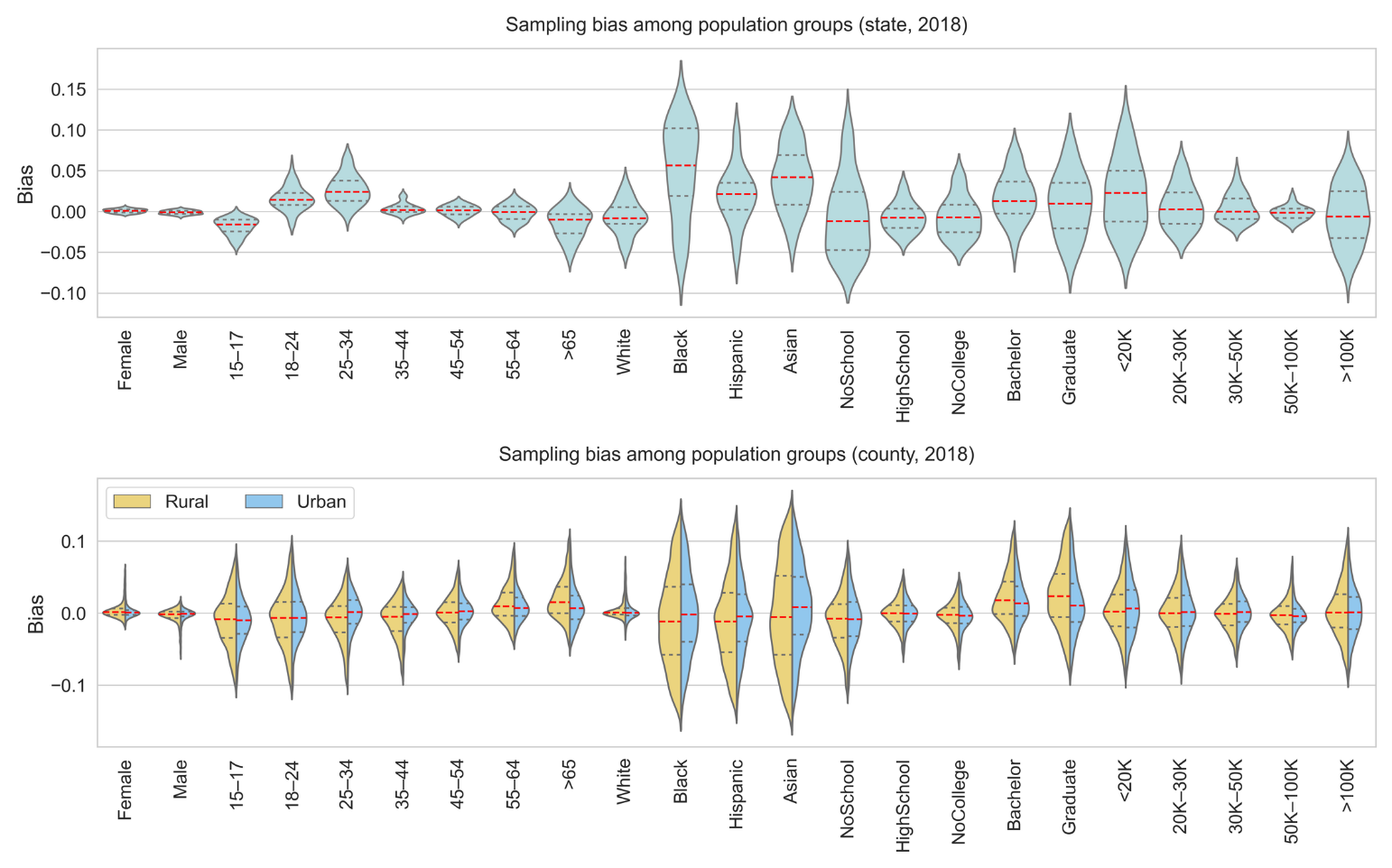


**Figure A21. Socioeconomic and demographic bias in 2018**


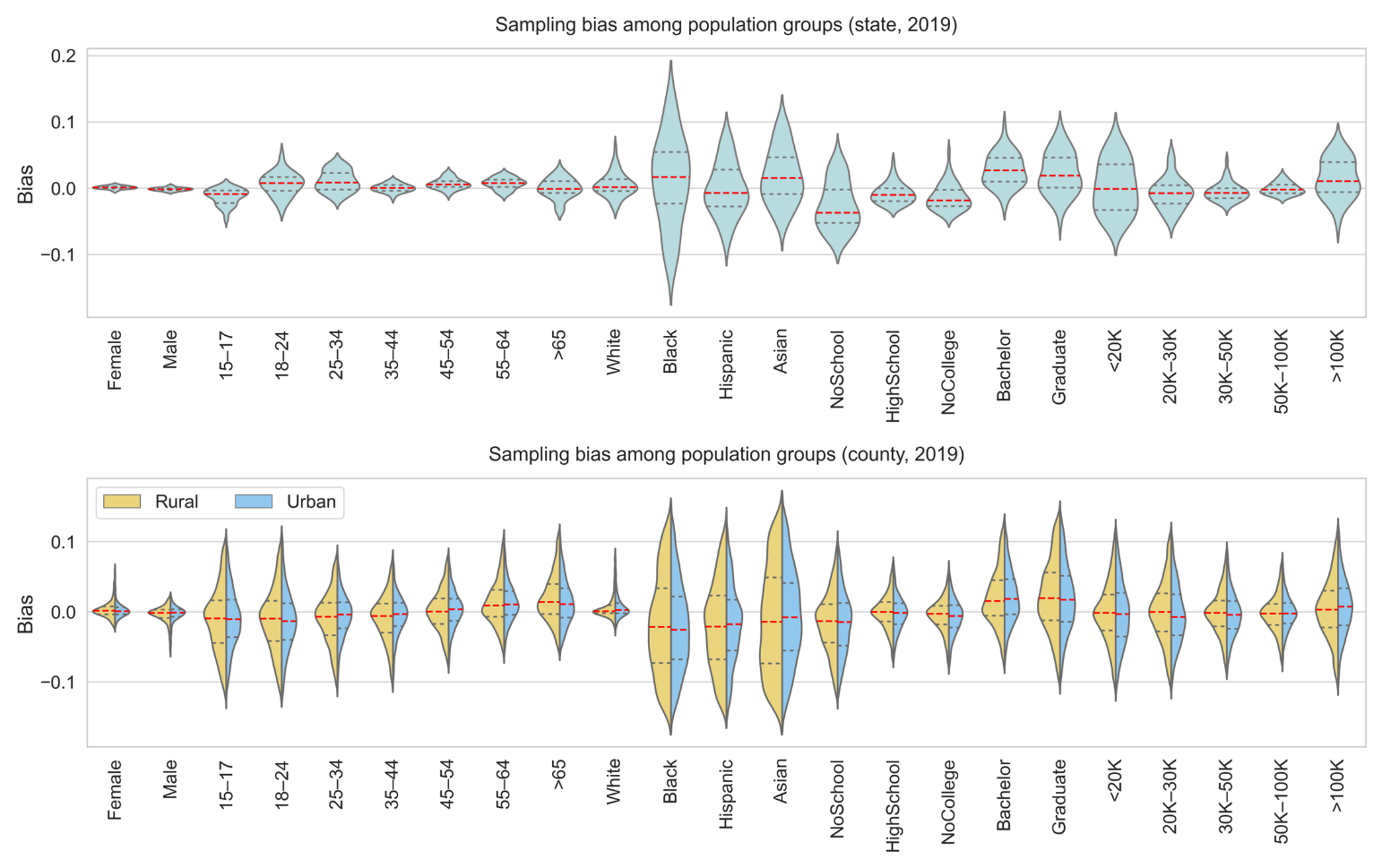


**Figure A22. Socioeconomic and demographic bias in 2019**


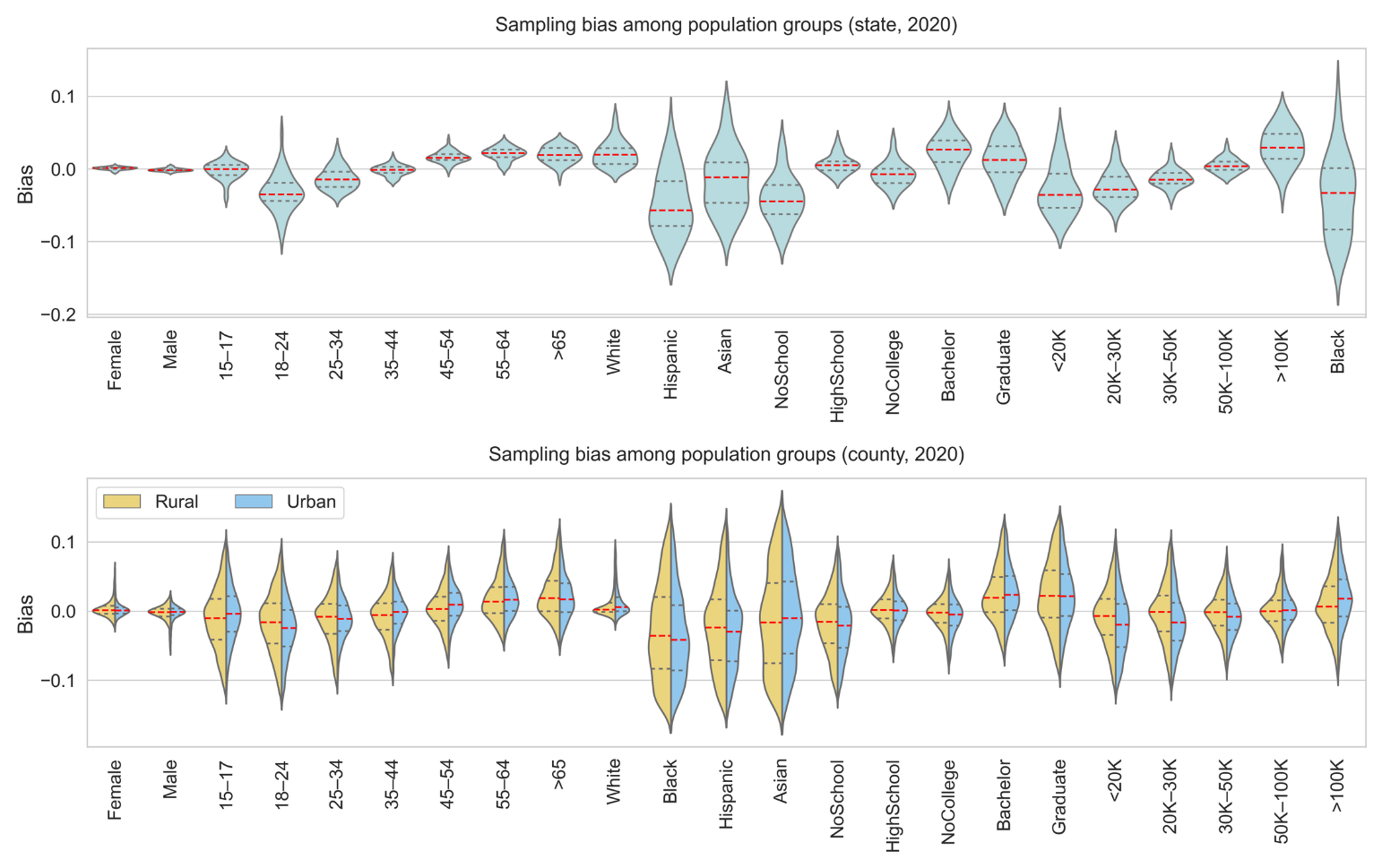


**Figure A23. Socioeconomic and demographic bias in 2020**


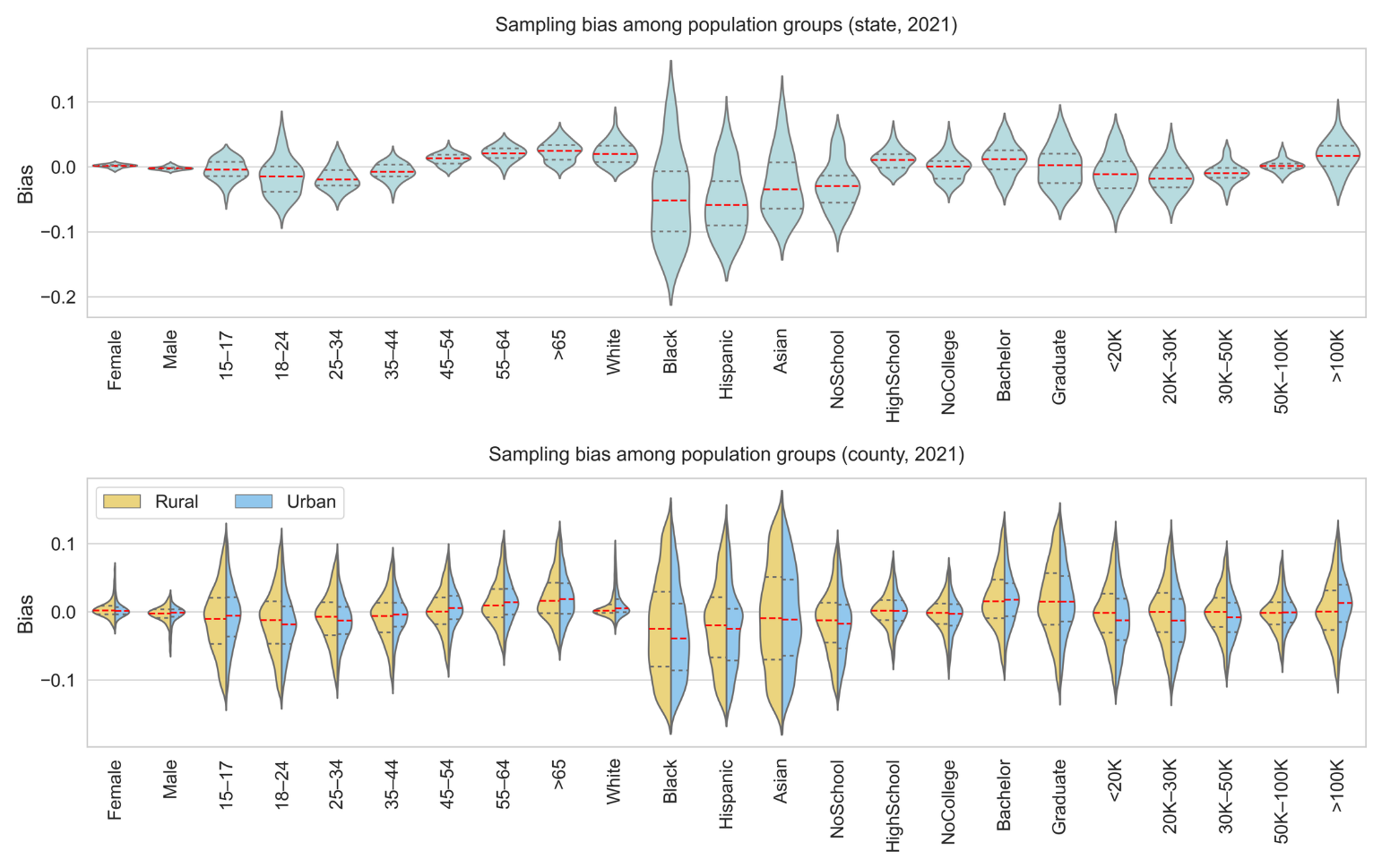


**Figure A24. Socioeconomic and demographic bias in 2021**


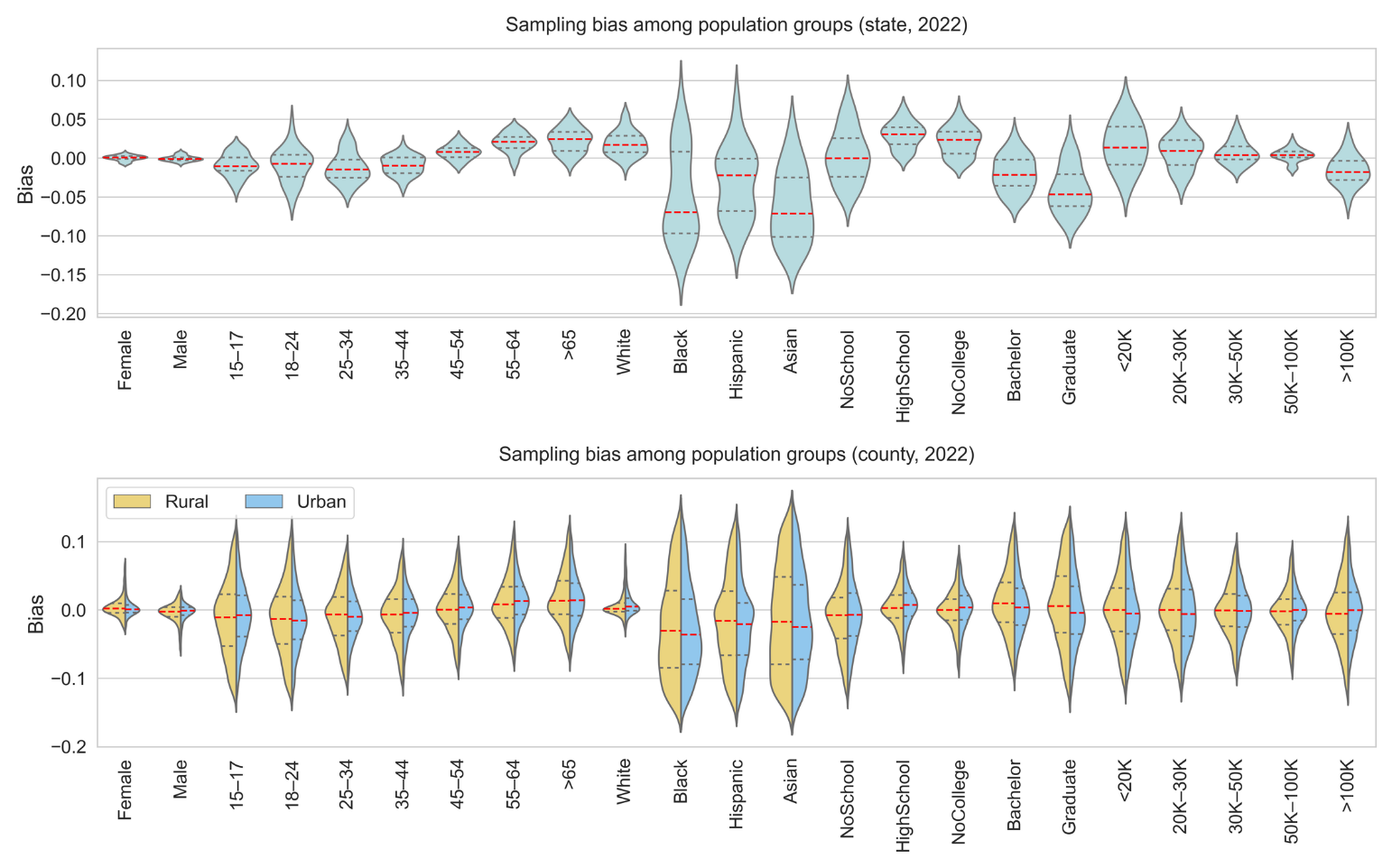


**Figure A25. Socioeconomic and demographic bias in 2022**


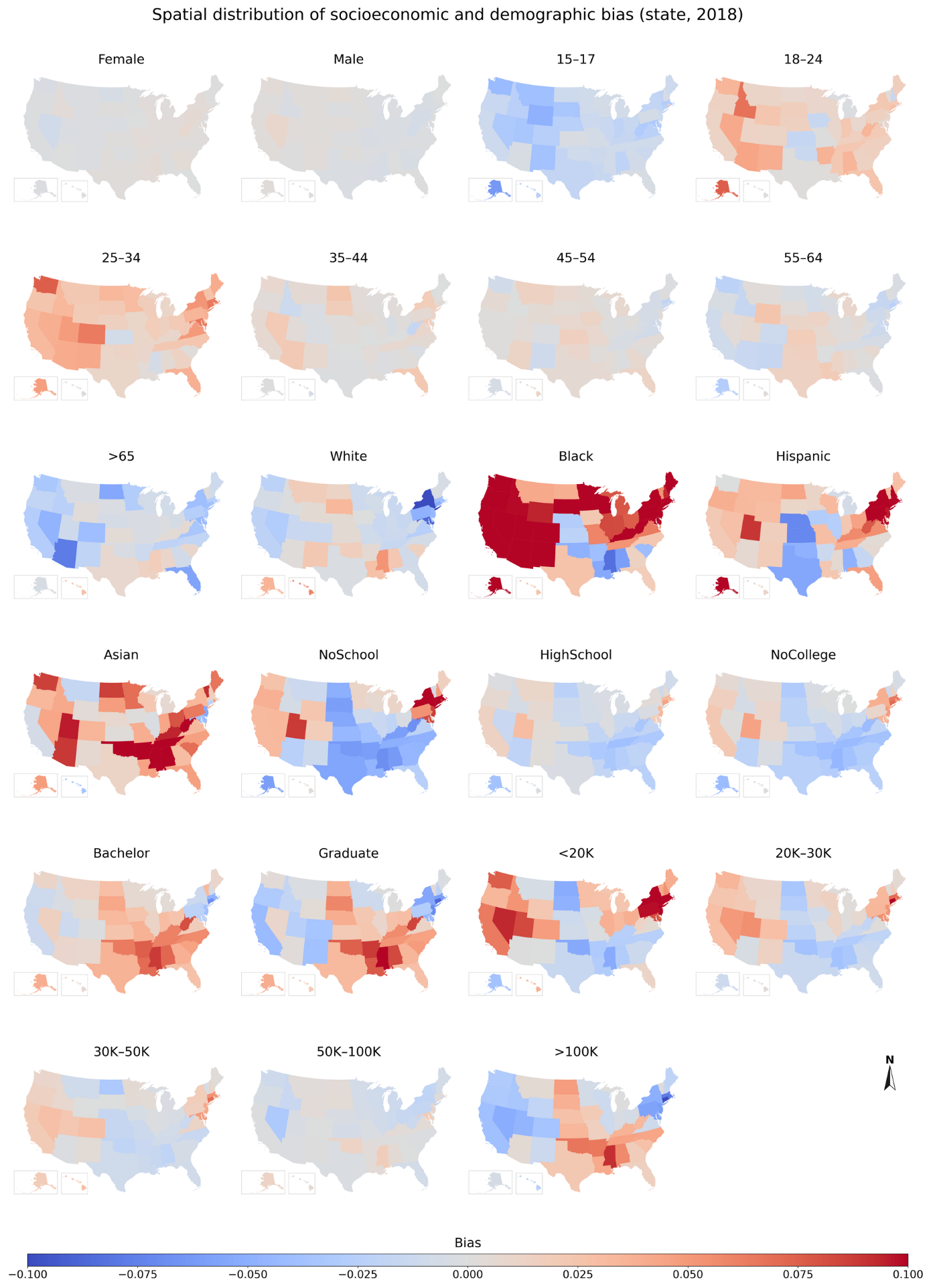


**Figure A26. Spatial distribution of socioeconomic and demographic bias at state level in 2018**


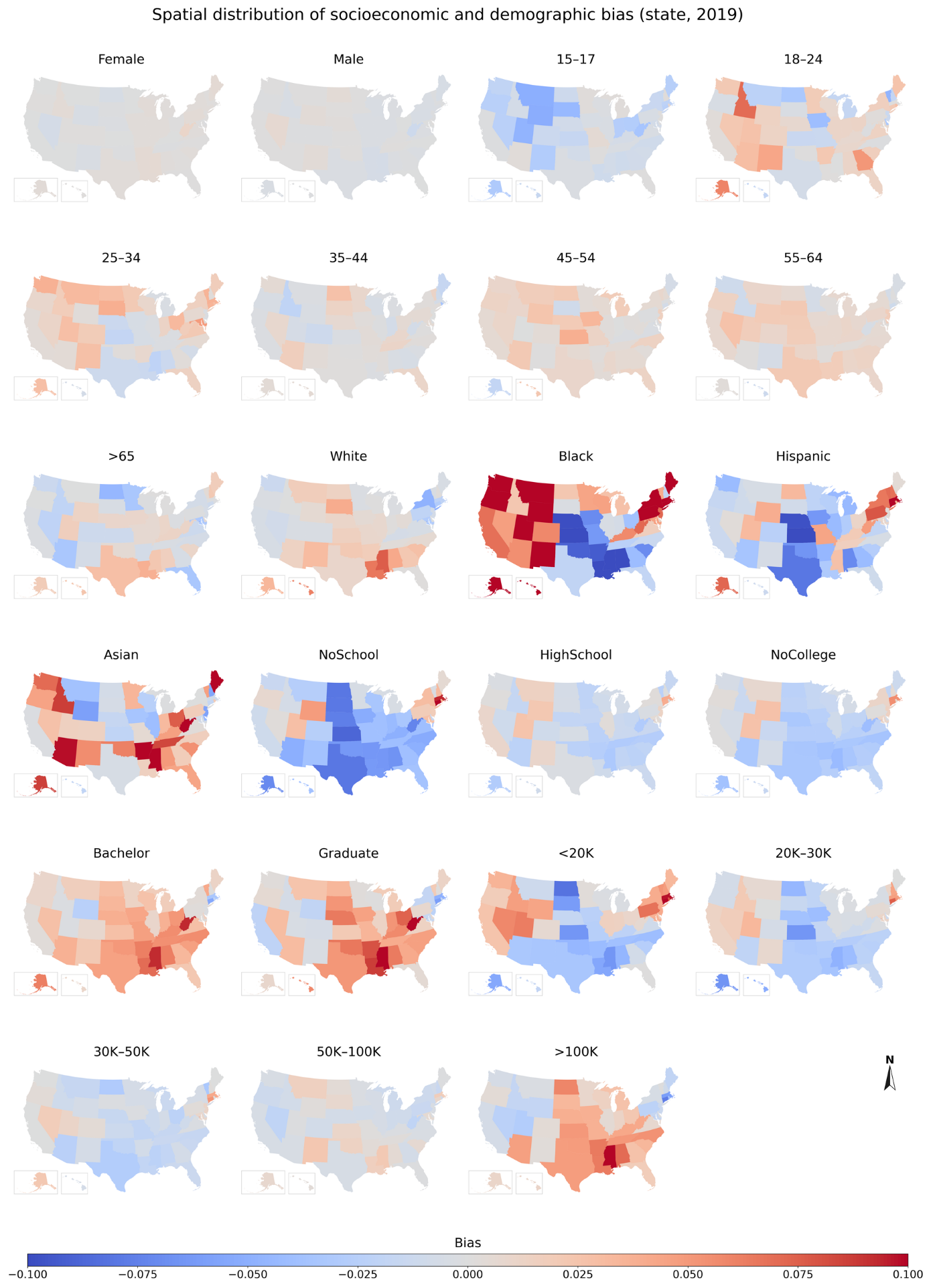


**Figure A27. Spatial distribution of socioeconomic and demographic bias at state level in 2019**


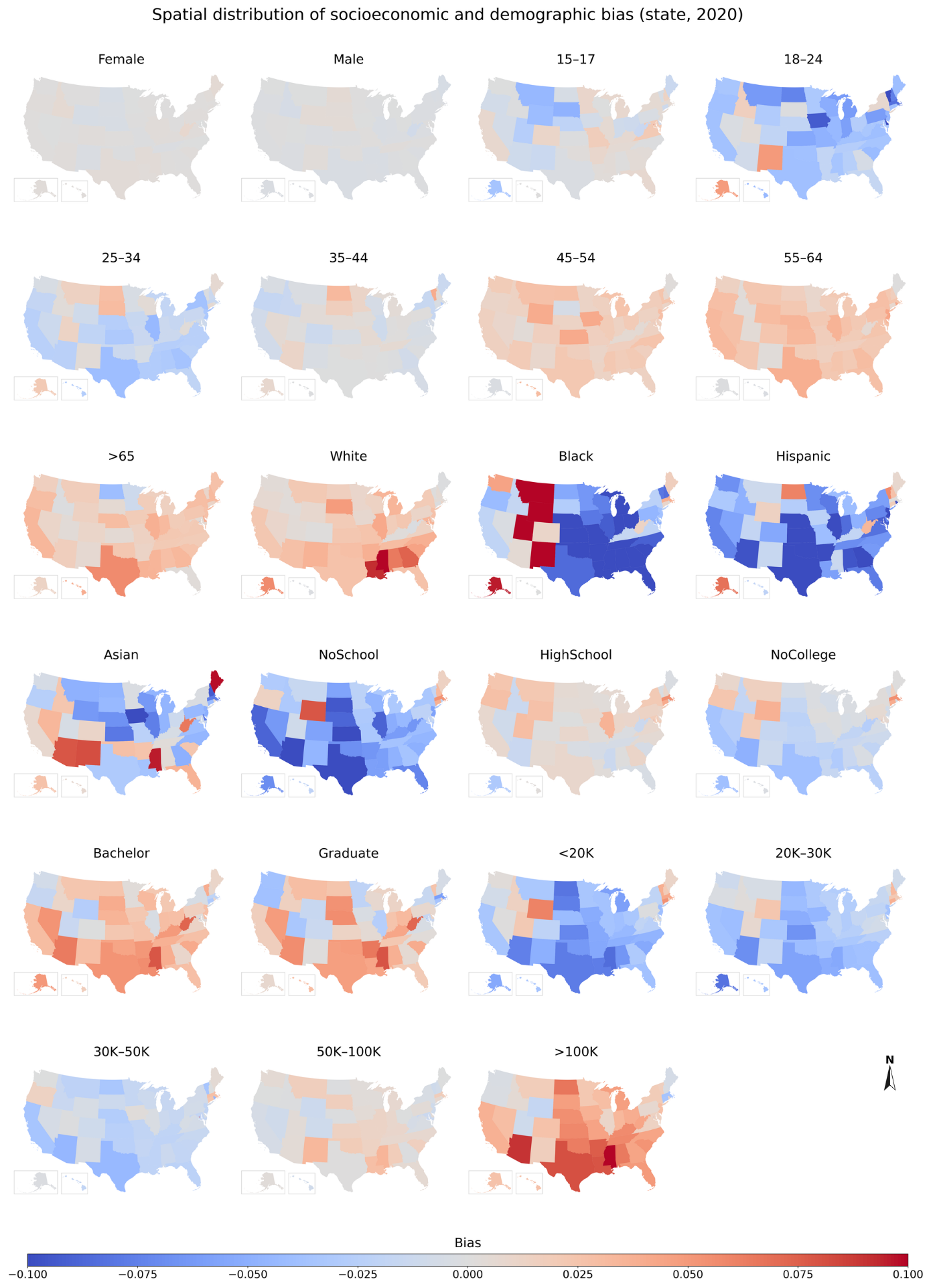


**Figure A28. Spatial distribution of socioeconomic and demographic bias at state level in 2020**


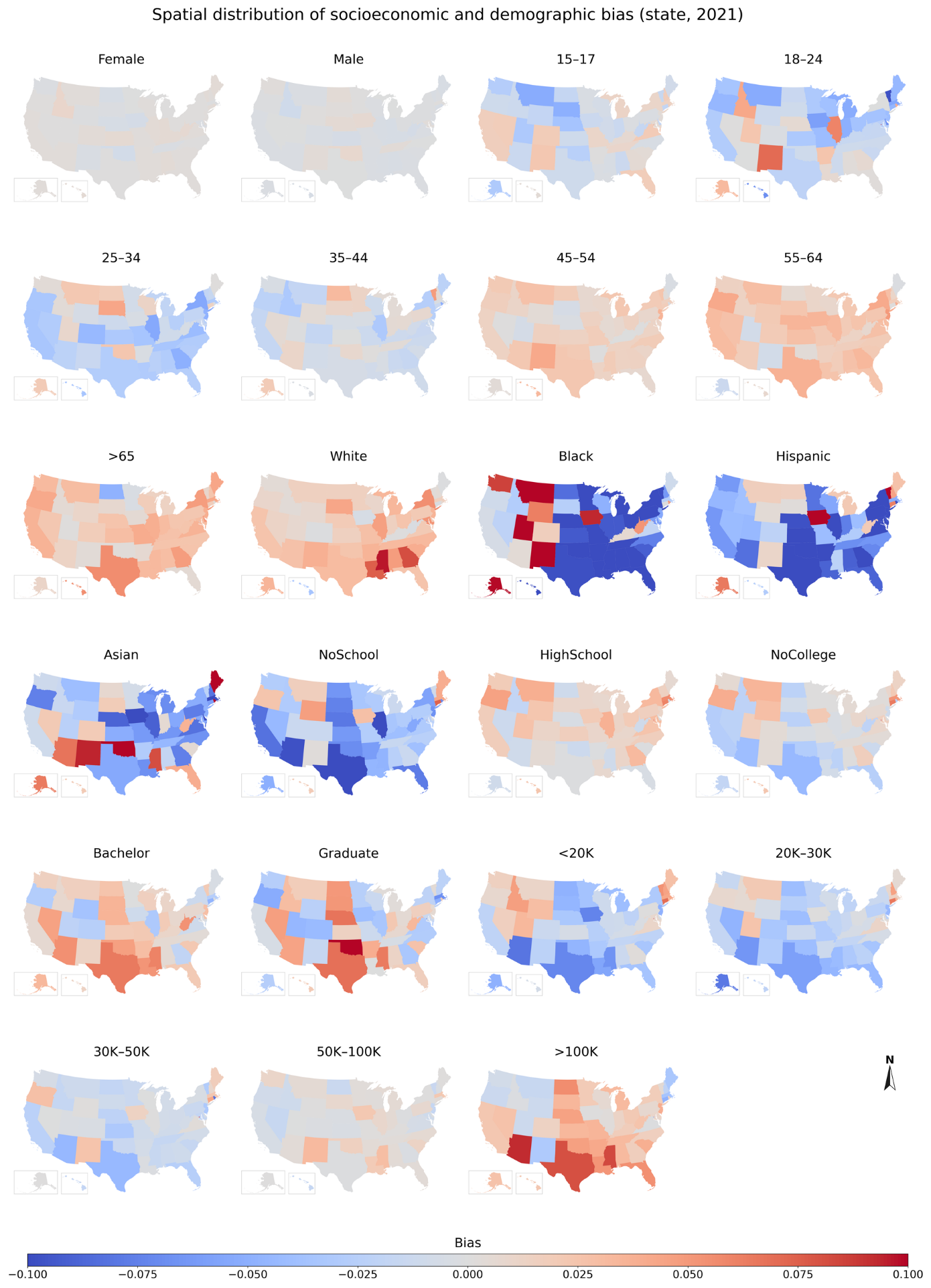


**Figure A29. Spatial distribution of socioeconomic and demographic bias at state level in 2021**


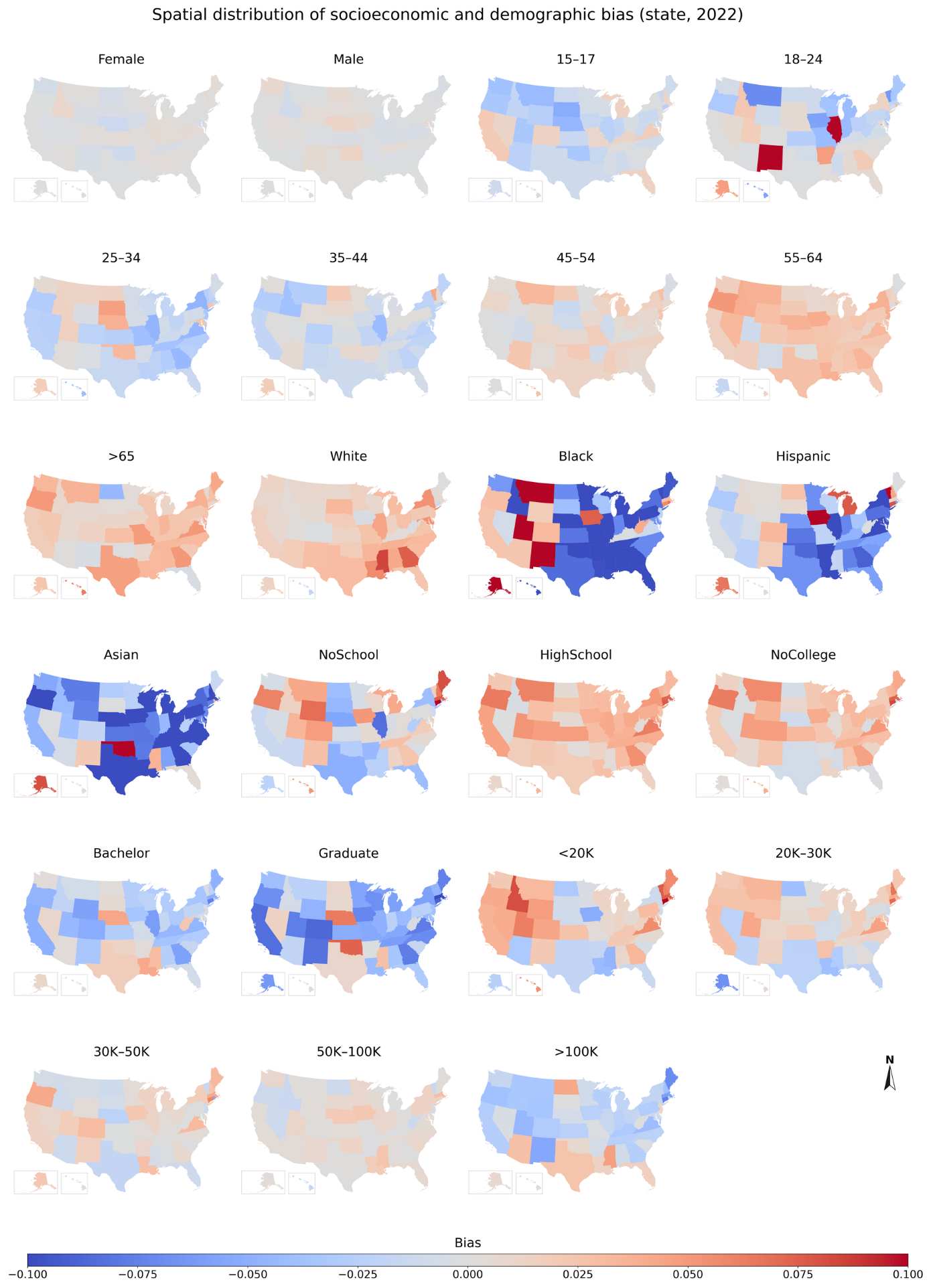


**Figure A30. Spatial distribution of socioeconomic and demographic bias at state level in 2022**


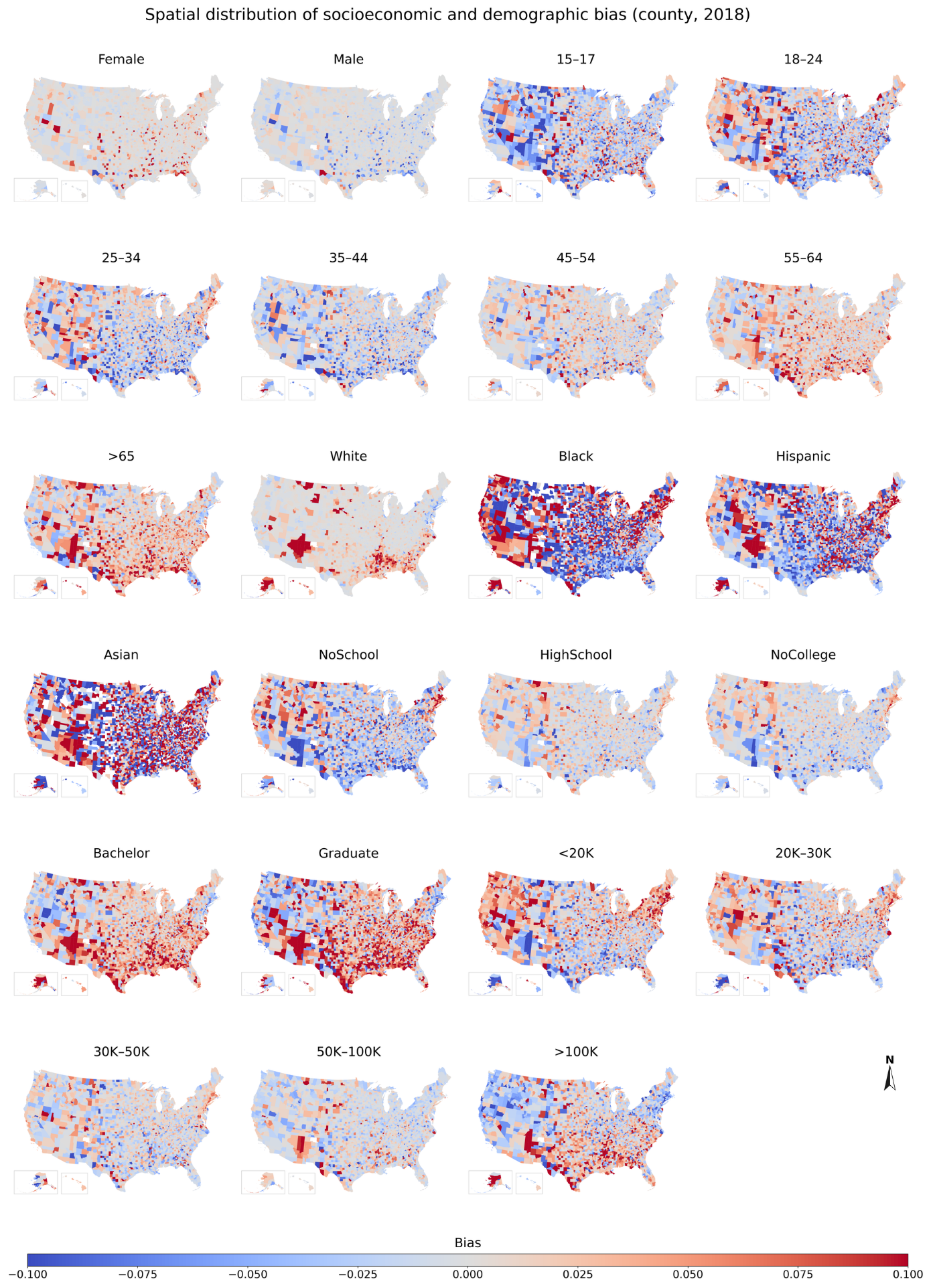


**Figure A31. Spatial distribution of socioeconomic and demographic bias at county level in 2018**


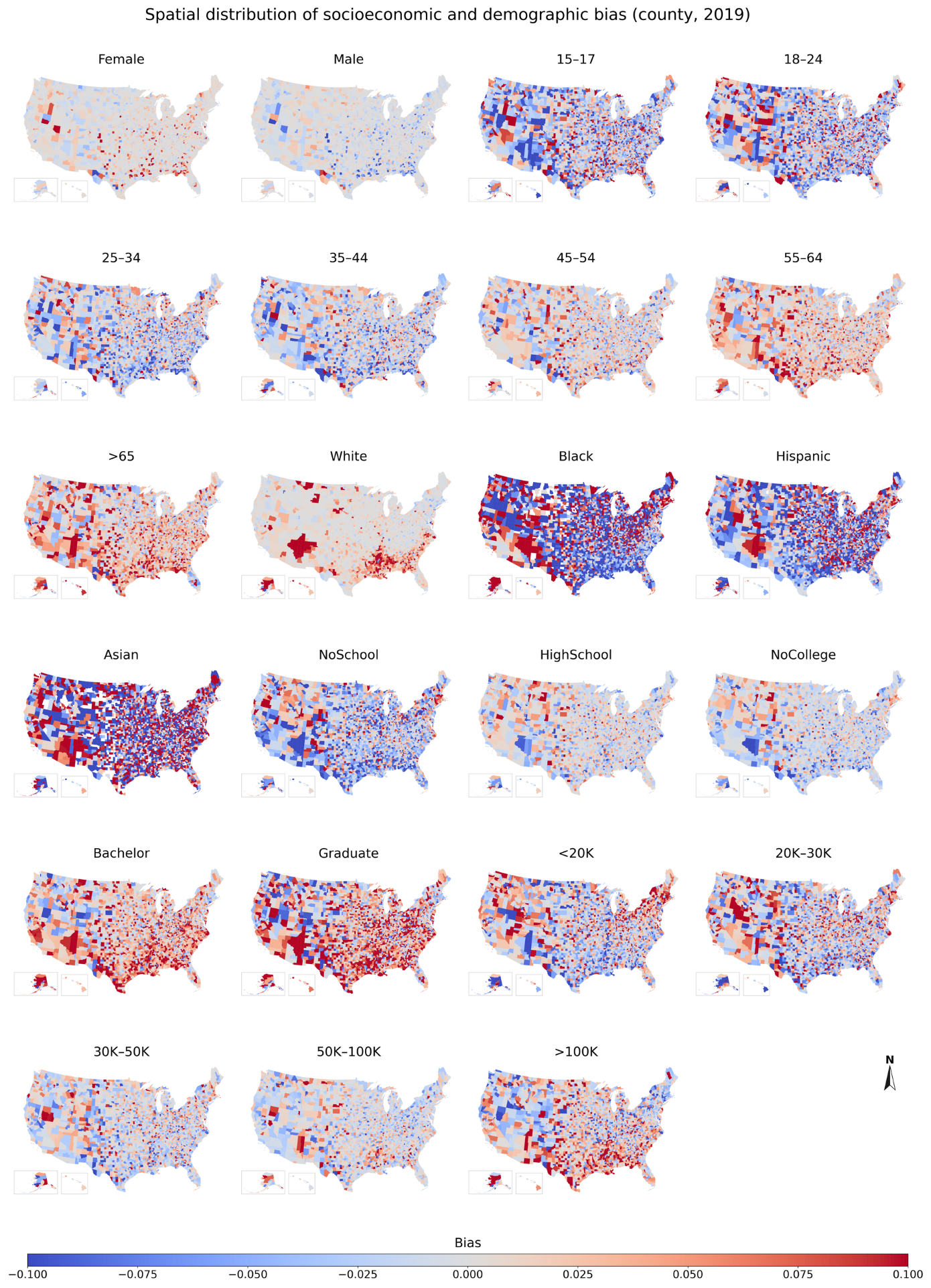


**Figure A32. Spatial distribution of socioeconomic and demographic bias at county level in 2018**


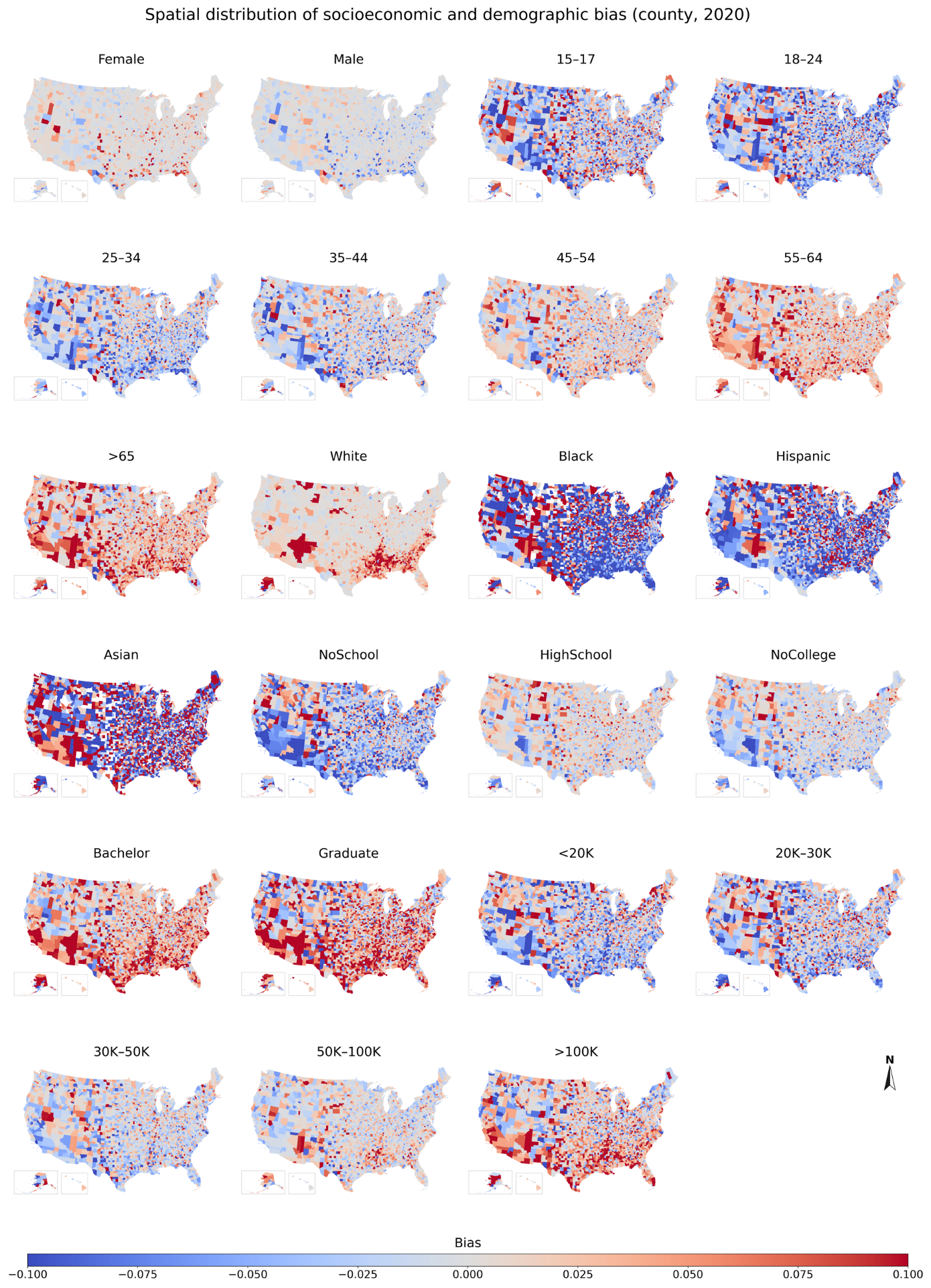


**Figure A33. Spatial distribution of socioeconomic and demographic bias at county level in 2020**


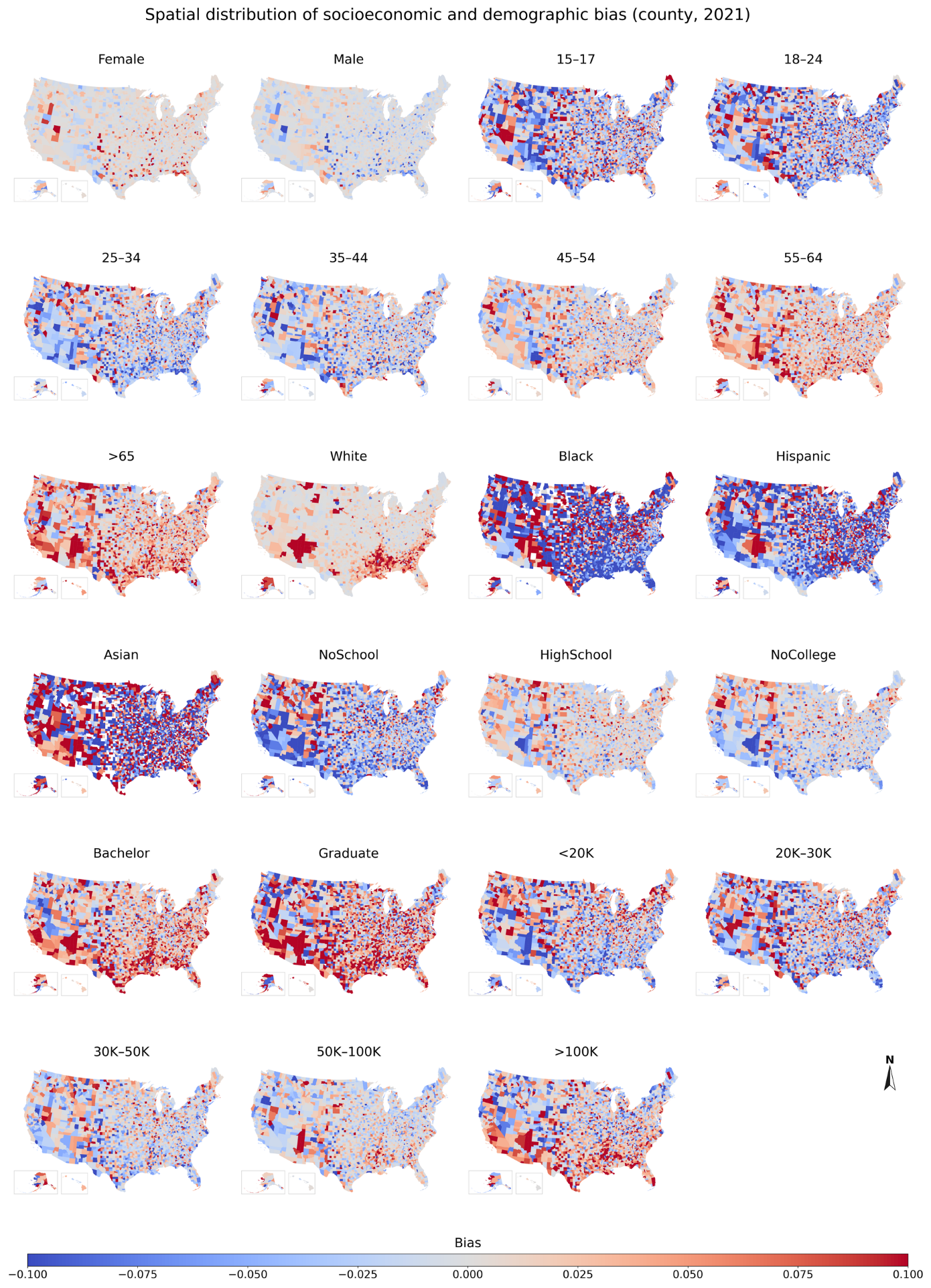


**Figure A34. Spatial distribution of socioeconomic and demographic bias at the county level in 2021**


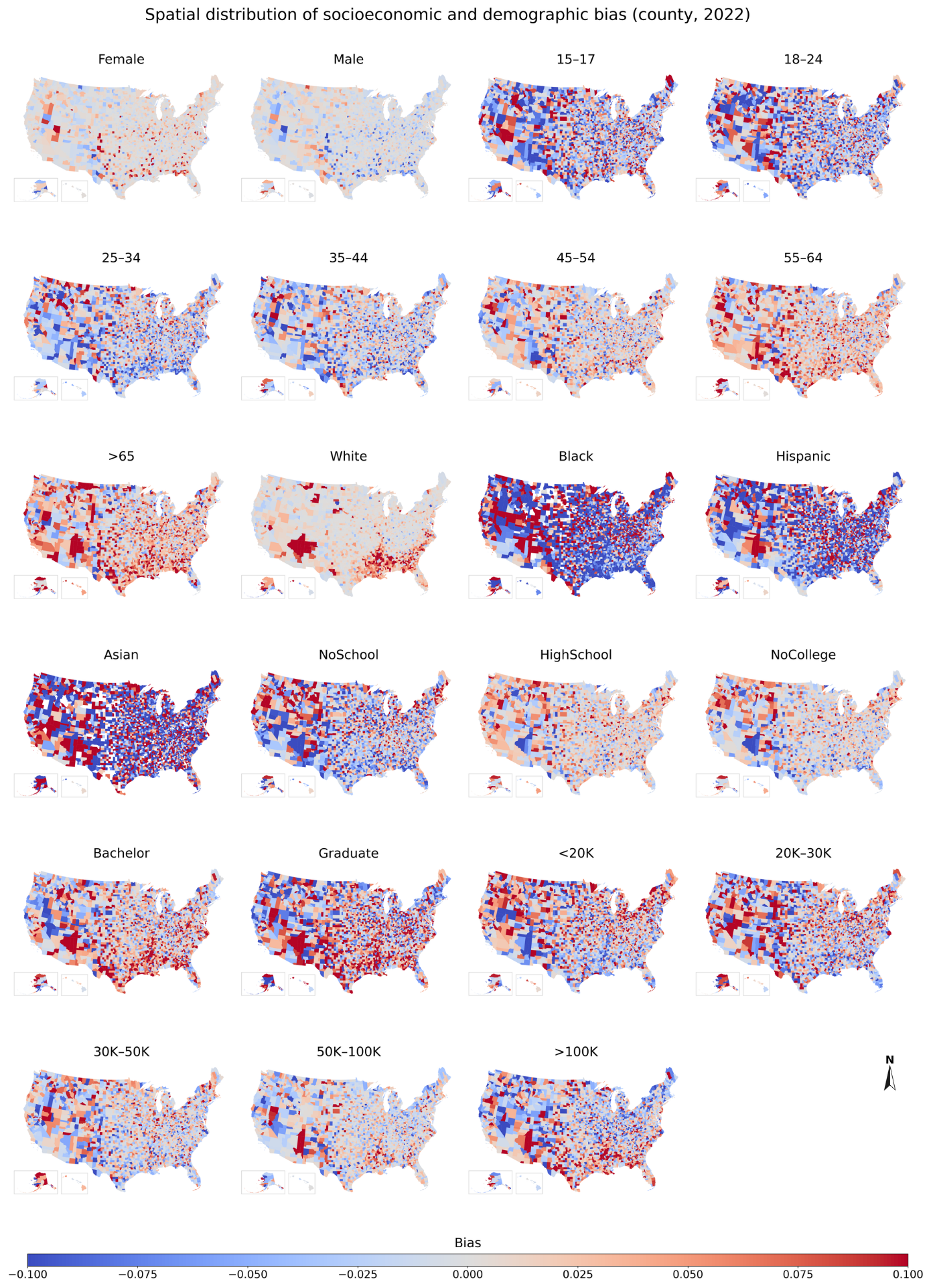


**Figure A35. Spatial distribution of socioeconomic and demographic bias at county level in 2022**
